# Genomic prediction of phytase potential and stress tolerance in maize-associated plant growth-promoting rhizobacterium *Enterobacter cloacae* Mz49

**DOI:** 10.1186/s13568-025-01981-8

**Published:** 2025-11-29

**Authors:** Mai A. Amer, Samira M. Hamed

**Affiliations:** Department of Microbiology and Immunology, Faculty of Pharmacy, October University for Modern Sciences and Arts (MSA), Giza, Egypt

**Keywords:** *Enterobacter cloacae*, *Zea mays*, PGPR, Stress tolerance, Phytase degradation, Heavy metal resistance

## Abstract

The maize rhizobacterium Enterobacter cloacae Mz49 exhibits diverse plant growth-promoting and stress-adaptive traits, indicating strong potential for bioinoculant development. Mz49 was isolated from the Egyptian rhizosphere, and demonstrated in vitro production of indole-3-acetic acid (64.89 µg/mL), antioxidant activity (IC₅₀ = 11.71 µg/mL), and anti-inflammatory properties (IC₅₀ = 63.1 µg/mL). Whole-genome sequencing revealed a 5.34 Mb genome with 5,158 protein-coding genes linked to nutrient acquisition, abiotic stress tolerance, and secondary metabolite biosynthesis. Genes associated with nitrogen fixation (nirBD, ureABCDEFGJ, amtB), phosphate solubilization (pqqIFL, phoA, pstIPN), and phytohormone synthesis (iaaT, ysnE, ipdC, ppdC, amiE) were identified. Genome analysis also revealed phytase-related genes (agp, suhB), which contribute to the release of bioavailable phosphorus, an essential nutrient for maize growth. This genetic potential highlights the strain’s contribution to enhanced plant growth and nutrient-use efficiency, particularly under phosphorus-limited conditions. Additionally, stress-response genes (sodABC, katE, betB, proABCY, dnaJK, cspA) were identified, conferring resistance to oxidative, osmotic, thermal, and cold stresses. The presence of heavy metal resistance genes (arsenic, copper, zinc, nickel) suggests suitability for use in contaminated soils. AntiSMASH analysis identified biosynthetic gene clusters for siderophores, arylpolyenes, and non-ribosomal peptides. GC–MS profiling further detected bioactive compounds, including 2, 3-butanediol, D-pinitol, succinic acid, tyrosol, and azelaic acid, which are associated with plant growth promotion and defense responses. Collectively, these findings highlight Mz49’s potential for sustainable maize cultivation, particularly in phosphorus-limited or stress-prone soils. Future research should prioritize field trials to validate its efficacy and assess potential risks associated with its application.

**Supplementary Information:**

The online version contains supplementary material available at 10.1186/s13568-025-01981-8.

## Introduction

Maize (*Zea mays* L.) is a staple food crop worldwide and a central model for studying plant–microbe interactions in the field of microbiology (Baloch, 2025). The microbiome associated with maize, particularly in the rhizosphere and endosphere, plays a crucial role in plant health, nutrient uptake, and stress tolerance. Microorganisms such as bacteria, fungi, and archaea in the maize microbiome can promote growth through nitrogen fixation, phosphorus solubilization, and the production of growth hormones like indole-3-acetic acid (IAA) (Trivedi et al. 2020). These beneficial microbes also enhance maize resistance to environmental stressors such as drought, pests, and diseases by improving soil health and activating plant defense mechanisms (Iqbal et al. 2023; Sharma et al. 2025). Advances in microbial genomics have revealed that plant growth-promoting rhizobacteria (PGPR) can also harbor uncharacterized metabolic pathways, highlighting their potential in improving nutrient cycling and supporting sustainable agriculture, revealing how microbial communities can be used to optimize crop production (Mehta et al. 2021; Gardner et al. 2022; Huang et al. 2022). This growing understanding of plant-microbiome interactions is paving the way for more sustainable agricultural practices (Compant et al. 2024).

Maize rhizobacteria play an important role in enhancing plant resilience against environmental stresses by promoting antioxidant activity. These beneficial bacteria colonize the root zone and help the plant overcome oxidative stress, which is typically induced by factors such as drought, salinity, and heavy metal contamination. The antioxidant activity of maize rhizobacteria is largely attributed to their ability to produce various enzymes and compounds, such as catalases, peroxidases, and superoxide dismutases, which neutralize reactive oxygen species (ROS) (Hasan et al. 2024). By reducing the levels of ROS, these rhizobacteria help maintain cellular integrity and improve the overall health and growth of maize plants. Maize-associated rhizobacterium *Azospirillum brasilense* enhances maize growth by fixing nitrogen, improving root development, and increasing drought tolerance through better water uptake (Hagh et al. 2010). *Pseudomonas fluorescens* aids in biological control by suppressing soil-borne pathogens through antibiotic production and resource competition (Ganeshan and Manoj Kumar, 2005; Prasad et al. 2019). *Enterobacter cloacae* is another important rhizobacterium associated with maize. It promotes plant growth by fixing nitrogen, solubilizing phosphate, and producing phytohormones, while also helping the plant tolerate abiotic stresses like salinity and drought (Ji et al. 2020; Chieb and Gachomo 2023; Sallam et al. 2024).

Among PGPR functions, phytase activity is of particular importance because it enables bacteria to hydrolyze phytate -an abundant but poorly available form of organic phosphorus in soils- into plant-accessible phosphate. The phytase potential of the *Enterobacteriaceae* family, particularly *Enterobacter* species, remains understudied despite their established roles in phosphate solubilization and plant growth promotion. Recent studies demonstrated that taxa such as *Klebsiella* and *Chryseobacterium* species harbor novel phytase genes and can metabolize phytate as a sole carbon and phosphorus source (Sajidan et al. 2004; Maldonado-Pava et al. 2024), suggesting similar capabilities may exist in *Enterobacter* but remain underexplored (Kalsi et al. 2016; Maldonado-Pava et al. 2024). Furthermore, *E. cloacae* has been reported to enhance maize growth by facilitating phosphorus acquisition and producing growth-promoting metabolites (Chen et al. 2022; El Ifa et al. 2024). These findings highlight the need to investigate phytase-related genes and enzymatic pathways in maize-associated *Enterobacter*, as this could unlock sustainable strategies for improving phosphorus bioavailability and reducing dependence on chemical fertilizers.

The genus *Enterobacter* consists of 63 recognized species (https://lpsn.dsmz.de/search?word=enterobacter). These species can be both pathogenic and beneficial, as some are associated with plant diseases and opportunistic infections in humans, while others are used in genetic engineering and as plant growth-promoting bacteria (Fadiji et al. 2023). Certain *Enterobacter* species also play crucial roles in biocontrol (Bonaterra et al. 2022). Understanding the roles of key regulators, including biochemical adaptations, genetic modifications, plant growth-promoting genes, and mechanisms for abiotic stress resistance, is crucial for advancing sustainable and eco-friendly agriculture. Equally important is the evaluation of biosafety aspects, particularly the presence of virulence or antibiotic resistance genes, to ensure safe application of potential bioinoculants. In this study, we report the genomic and functional characterization of *E. cloacae* Mz49, a maize-associated PGPR with phytase activity and stress-adaptive traits. We combined genome sequencing, in vitro assays, antiSMASH analysis, and GC–MS profiling to assess its potential as a safe and effective bioinoculant for sustainable agriculture. Therefore, this study presents the first genomic report on *E. cloacae* isolated from the maize rhizosphere in Egypt.

## Materials and methods

### Rhizobacteria isolation and cultivation

The *E. cloacae* strain Mz49 was isolated from the rhizosphere of maize (*Zea mays* L.) in agricultural fields located in Giza Governorate, Egypt (coordinates: 30° 0′ 47.0016'' N, 31° 12′ 31.8708'' E). The soil in this region is predominantly clay, with moderate levels of sand and silt, and is generally alkaline with a neutral to high pH. It is also characterized by favorable physical properties and high water-holding capacity (El-Sherpiny et al. 2020). Soil samples were collected in sterile bags and transported to the lab on ice. In the laboratory, 1 g of soil was suspended in 20 ml of sterile saline and incubated at 30°C with shaking at 200 rpm for 30 min. Serial dilutions were plated on Nutrient Agar (pH 7.0) and incubated at 30 °C for 48 h. Distinct colonies were then selected for further purification and were subsequently maintained on sterile nutrient agar slants at 4 °C for additional analysis (Amer et al. 2021). The preliminary identification of the isolate involved Gram staining, oxidase test, and culturing on differential media such as MacConkey’s agar and Triple Sugar Iron (TSI) agar (Murray and Baron 2007).

### Preparation of the cell-free supernatant (CFS)

To prepare the bacterial CFS, an overnight culture of rhizobacteria grown in Trypticase Soy Broth (TSB) was adjusted to an OD_600_ of 1. This adjusted culture was then diluted 1:100 in fresh TSB media. The flasks were then incubated at 30 °C with shaking at 120 rpm for 4 days. The CFS was collected by centrifuging the culture at 10,000 rpm for 10 min at 4 °C, followed by filtration through a 0.22 μm filter (Millipore, Bedford, MA, USA) (Amer et al. 2023).

Functional assays were conducted on the CFS to characterize traits relevant to plant growth promotion and stress inhibition. IAA production was assessed as it plays a crucial role in stimulating root elongation and enhancing nutrient acquisition in host plants (Salem et al. 2024). Antioxidant activity was evaluated to determine the ability of strain Mz49 to scavenge free radicals and reduce oxidative damage commonly associated with abiotic stress (Sun et al. 2024). Anti-inflammatory activity was tested to explore potential interactions between bacterial metabolites and plant defense pathways, that may inhibit inflammation-related stress and promote overall plant health (Khadem et al. 2014; Ruiz-Santiago et al. 2025).

### Determination of IAA production

For assessing the IAA production potential of Mz49, the CFS was prepared as described above, but using TSB medium supplemented with L-tryptophan (100 mg/l). The concentration of IAA in the CFS was measured using the Salkowski colorimetric assay as described before (Glickmann and Dessaux 1995; Amer et al. 2021). Briefly, 1 ml of the CFS was combined with 2 mL of Salkowski’s reagent and incubated in the dark at room temperature for 30 min. The appearance of a pink coloration confirmed IAA production, and optical density (OD) was measured at 530 nm using a UV1800 spectrophotometer (Shimadzu, Japan) (Patten and Glick 2002; Aziz et al. 2015). A negative control, consisting of uninoculated medium treated with Salkowski’s reagent, was processed in parallel. A standard curve was prepared using IAA concentrations ranging from 0 to 100 μg/ml. Absorbance values were converted to IAA concentrations using the standard curve, and results were expressed as the mean of three independent replicates.

### Evaluation of antioxidant activity of Mz49 via DPPH radical scavenging

The free radical scavenging activity of Mz49 CFS was assessed using the 1, 1-diphenyl-2-picryl hydrazyl (DPPH) method. A 0.1 mM DPPH solution in ethanol was prepared, and 1 ml of this solution was mixed with 3 ml of the CFS at different concentrations (0.2–100%). The mixture was shaken vigorously and left to stand at room temperature for 30 min, after which absorbance was measured at 517 nm using a UV–VIS spectrophotometer (Milton Roy). Ascorbic acid was used as the reference standard, and the experiment was conducted in triplicate. The IC_50_ value, representing the concentration of the sample required to inhibit 50% of DPPH free radicals, was determined using a Log dose inhibition curve. Lower absorbance of the reaction mixture indicated higher free radical scavenging activity (González-Palma et al. 2016; Baliyan et al. 2022). The percentage of DPPH scavenging effect (or percent inhibition) was calculated using the formula:

$$ Inhibition percentage \left( \% \right) = \frac{A0 - A 1 }{{A0 }} *100 $$

where A0 was the Absorbance of the control reaction, and A1 was the Absorbance in the presence of the test or reference standard.

### Evaluation of the anti-inflammatory and membrane stabilization activity of Mz49

#### Preparation of erythrocyte suspension

Fresh whole blood (3 ml) was collected in heparinized tubes and centrifuged at 3000 rpm for 10 min. The red blood cell pellet was resuspended in an equal volume of normal saline to replace the removed supernatant. The suspension was then adjusted to a 40% (v/v) concentration using an isotonic 10 mM sodium phosphate buffer (pH 7.4). The buffer consisted of 0.2 g NaH₂PO₄, 1.15 g Na₂HPO₄, and 9 g NaCl per liter of distilled water. The reconstituted red blood cells were subsequently used for analysis (Gunathilake et al. 2018).

### Hypotonicity-induced hemolysis

The CFS was dissolved in distilled water to create a hypotonic solution. For each concentration ranging from (0.4–100%), 5 ml of this hypotonic solution was placed into duplicate centrifuge tubes. Similarly, 5 ml of isotonic solution with CFS concentrations ranging from (0.4–100%) were also placed into duplicate tubes. Control tubes contained 5 ml of distilled water and 5 ml of indomethacin, respectively. To each tube, 0.1 ml of erythrocyte suspension was added and mixed gently. The mixtures were incubated at room temperature (37 °C) for 1 h, then centrifuged at 1300 g for 3 min. The absorbance of the hemoglobin content in the supernatant was measured at 540 nm using a spectrophotometer. Hemolysis percentage was calculated with the assumption that hemolysis in the presence of distilled water was 100% (Shinde et al. 1999; Anosike et al. 2012). The IC_50_ value, representing the concentration of the sample required to inhibit 50% of haemolysis, was determined using a Log dose inhibition curve. The percent inhibition of hemolysis by the Mz49 CFS was calculated using the formula:

$$ Inhibition of haemolysis \left( \% \right) = 1 - \frac{OD2 - OD1 }{{OD3 - OD1}}*100 $$

where OD1 = absorbance of test sample in isotonic solution, OD2 = absorbance of test sample in hypotonic solution, OD3 = absorbance of control sample in hypotonic solution.

#### Genome analysis using whole-genome sequencing

##### Library preparation and sequencing

DNA from strain Mz49 was extracted using the QIAamp® DNA Mini Kit (QIAGEN, Germany). Library preparation and Whole Genome Sequencing (WGS) were carried out by BGI Tech Solutions in Tai Po, Hong Kong, China, using the DNBseq™ sequencing platform developed by BGI. Quality control and trimming of reads before assembly were performed with BGI's SOAPnuke software, ensuring the production of high-quality sequencing data for further analysis and interpretation (Chen et al. 2018).

#### Genome assembly and annotation

The pre-processed reads were assembled de novo using SPAdes v. 2.1, following the method described by Wick et al. (2017). The resulting contigs were annotated with the Prokaryotic Genome Annotation Pipeline (PGAP) from the National Center for Biotechnology Information (NCBI). The annotated genome was then deposited in GenBank for public access (http://www.ncbi.nlm.nih.gov/genome/annotation_prok).

The functional annotation of predicted proteins was performed using three primary databases. ORFs were annotated through BLAST searches against the UniProtKB and Kyoto Encyclopedia of Genes and Genomes (KEGG) databases. Analysis of the circular chromosome and GC skew was carried out using the CGViewer Server (Grant and Stothard 2008), and the Proksee server (Grant et al. 2023), respectively. For subsystem distribution, the SEED-based annotation was achieved via the Rapid Annotations using Subsystems Technology (RAST) server. COG categories were assigned following the methodology of Aziz et al. (2008) and further analyzed with eggNOG-mapper using the NCBI COG database (Cantalapiedra et al. 2021). Additionally, KEGG Orthology (KO) assignments were also completed using GhostKOALA, based on the approach by Kanehisa et al. (2016). These integrated analyses provided comprehensive insights into the gene functions and pathways within the genome.

### Phylogenomic analysis and genome-based taxonomy

To explore the taxonomy of strain Mz49, its draft genome sequence was examined using various genome-based taxonomy tools. The Type Strain Genome Server (TYGS) by DSMZ was employed to identify the closest type strain genomes (https://tygs.dsmz.de/) (Meier-Kolthoff and Goker 2019).

The taxonomic position of Mz49 was further assessed by calculating the overall genome-relatedness indices (OGRI) between Mz49 and other closely related species (Chun and Rainey 2014; Riesco and Trujillo 2024). The JSpeciesWS tool was utilized to calculate the average nucleotide identity (ANI) using the BLAST + alignment algorithm (ANIb) and MUMmer (ANIm), as well as the Tetra-nucleotide signature correlation index (Tetra) (Richter et al. 2015). Digital DNA–DNA hybridization (dDDH) values were determined using the Genome-to-Genome Distance Calculator 3.0, available at (https://ggdc.dsmz.de/ggdc.php) (Meier-Kolthoff et al. 2021).

**Table 1:** Functional analysis of the predicted protein products of Mz49 according to KEGG and COG databases

A. KEGG pathway functional class (complete modules)	CDS	CDS%	B. COG functional class	CDS	CDS%
– Carbohydrate metabolism	163	28.50%	C—Energy production and conversion	304	6.67%
– Energy metabolism	53	9.27%	E—Amino acid transport and metabolism	297	6.52%
– Lipid metabolism	22	3.85%	F—Nucleotide transport and metabolism	134	2.94%
– Nucleotide metabolism	46	8.04%	G—Carbohydrate transport and metabolism	270	5.93%
– Amino acid metabolism	127	22.20%	H—Coenzyme transport and metabolism	276	6.06%
– Glycan biosynthesis and metabolism	19	3.32%	I—Lipid transport and metabolism	117	2.57%
– Metabolism of cofactors and vitamins	104	18.18%	P—Inorganic transport and metabolism	338	7.42%
– Metabolism of terpenoids and polyketides	14	2.45%	Q—Secondary metabolites biosynthesis	58	1.27%
– Biosynthesis of other secondary metabolites	4	0.70%	D—Cell cycle control, cell division, chromosome partitioning	58	1.27%
– Xenobiotic biodegradation and metabolism	11	1.92%	M—Cell wall/membrane/envelope biogenesis	337	7.40%
– Signature modules	9	1.57%	N—Cell motility	188	4.13%
			O—Post-translational modification, protein	98	2.15%
			T—Signal transduction mechanisms	124	2.72%
			U—Intracellular trafficking, secretion, and vesicular transport	57	1.25%
			V—Defense mechanisms, information storage and processing	55	1.21%
			J—Translation, ribosomal structure and biogenesis	209	4.59%
			K—Transcription	457	10.03%
			L—Replication, recombination and repair	184	4.04%
			S—Function unknown	995	21.84%
Total	572	100%	Total	4556	100%

To assess the genomic similarity with closely related strains of the same species, we searched for and downloaded relevant genomes from the Bacterial and Viral Bioinformatics Resource Center database (https://www.bv-brc.org/) (Olson et al. 2023). The metadata of the strains included in the phylogenomic analysis of Mz49 are listed in Supplementary Table. 1. A whole-genome -based phylogenetic tree was created using the Codon tree tool hosted by BV-BRC and was visualized using the Interactive Tree of Life (iTOL) online tool, version 6.7 (https://itol.embl.de/itol.cgi).

The multilocus sequence types (MLST) of the isolates were determined using the PubMLST database for *Enterobacter* species (https://pubmlst.org/organisms/enterobacter-spp). This database applies the MLST scheme described by Miyoshi-Akiyama et al. (2013), which is based on seven housekeeping genes: *dnaA*, *fusA*, *gyrB*, *leuS*, *pyrG*, *rplB*, and *rpoB*.

### Identification of putative biosynthetic gene clusters (BGCs)

The antiSMASH server was employed to identify BGCs responsible for producing secondary metabolites within the bacterial genome (Blin et al. 2019). This tool utilizes a rule-based approach to detect various biosynthetic pathways involved in secondary metabolite production. It offers a detailed analysis of specific BGC classes, including non-ribosomal peptide synthetases (NRPSs), type I and II polyketide synthases (PKSs), and ribosomally synthesized and post-translationally modified peptides (RiPPs).

#### Screening of plant-related genes

The PGPT-Pred tool available through PLaBAse (https://plabase.cs.uni-tuebingen.de/pb/form.php?var=PGPT-Pred) is specifically designed for analyzing genes of bacterial plant growth-promoting traits (Hamed and Amer 2025). It predicts plant-associated bacteria by annotating approximately 123 proteins involved in plant interactions, utilizing the blastp + hmmer method. The tool is built on a machine learning model initially developed by Martínez-García et al. (2016).

#### Pathogenicity prediction and identification of antimicrobial resistance (AMR) and virulence genes

The pathogenic potential of Mz49 for human hosts was predicted using PathogenFinder, developed by Cosentino et al. (2013), which assigns a probabilistic score for pathogenicity (https://cge.cbs.dtu.dk/services/PathogenFinder/). The assembled genome was screened for antimicrobial resistance genes (ARGs) using ResFinder 4.1, with minimum length and threshold criteria set at 60 and 90%, respectively (https://cge.cbs.dtu.dk/services/ResFinder/), and the Comprehensive Antibiotic Resistance Database (CARD) (https://card.mcmaster.ca/analyze/rgi) (Alcock et al. 2020), using the "perfect and strict hits only" option. This multi-platform approach mitigated the limitations of using a single tool.

Virulence genes were identified using VirulenceFinder 2.0, with minimum length and threshold settings of 60 and 90%, respectively (https://cge.cbs.dtu.dk/services/VirulenceFinder/) (Joensen et al. 2014). Additionally, virulence factors were screened through the Virulence Factor Database (VFDB) (http://www.mgc.ac.cn/cgi-bin/VFs/v5/main.cgi?func=VFanalyzer) and BacWGSTdb (http://bacdb.cn/BacWGSTdb), focusing on virulence determinants specific to *E. cloacae*.

#### Metabolic characterization of Mz49 CFS using gas chromatography-mass spectrometry (GC–MS) analysis

Metabolites in the CFS were extracted using ethyl acetate following the method described by Amer et al. (2023). Five milligrams of the dried extract were combined with 120 μl of the silylating agent N,O-Bis(tert-butyl dimethyl silyl) acetamide and incubated at 60 °C for 30 min prior to analysis by gas chromatography (Wasfi et al. 2023). Gas chromatography analysis was conducted using a Shimadzu® GC system equipped with an AQP2010 Rtx-5MS column. Helium was used as the carrier gas, and 10 μl of the sample was injected. The analysis was performed over 45 min with a flow rate of 1.24 ml/min, while the oven temperature was programmed to rise from 60 to 260 °C. The resulting data were analyzed by comparing the mass spectra and retention indices of the detected peaks with entries in the National Institute of Standards and Technology (NIST) library.

#### In silico characterization of the phytase enzyme encoded by the *agp* gene

The functional annotation of the predicted phytase protein was further studied using several databases. BLASTp searches were done against the NCBI and UniProtKB databases. Multiple sequence alignments (MSA) of the predicted amino acid sequences were generated using Clustal Omega (version 1.2.4), available through the European Bioinformatics Institute (EMBL-EBI) at https://www.ebi.ac.uk/Tools/msa/clustalo/. The resulting alignments were visualized in color using MView (version 1.63), a multiple alignment viewer provided by EMBL-EBI at https://www.ebi.ac.uk/Tools/msa/mview/. A protein phylogenetic tree was constructed using MEGA11 using the maximum likelihood method (Tamura et al. 2013). ExPASy ProtParam was used to analyze the physicochemical properties of the protein. Conserved domains and catalytic core positions were searched using the NCBI Conserved Domains database https://www.ncbi.nlm.nih.gov/Structure/cdd/ (Wang et al. 2023). Expasy prosite https://prosite.expasy.org/, InterProScan https://www.ebi.ac.uk/interpro/, and HMMER (Pfam) https://www.ebi.ac.uk/Tools/hmmer/ were used to scan the signature domain in the protein sequence (Sigrist et al. 2013).

3D structure prediction was carried out using Phyre2 (Protein Homology/analogY Recognition Engine v2) in intensive mode (default parameters). The query sequence [413 aa] was submitted to the Phyre2 web server (http://www.sbg.bio.ic.ac.uk/phyre2). Phyre2 constructs hidden Markov models (HMMs) of the query and searches against its curated PDB fold library to identify homologous templates, followed by alignment-guided modelling. Per-residue secondary structure and disorder propensities reported by Phyre2 were used to annotate helices/strands and highlight potentially flexible regions that may affect model reliability (Kelley et al. 2015).

Protein–protein interaction networks were retrieved from STRING (version [12.0]) https://string-db.org/. Interactions with a combined score ≥ 0.7 were retained. Networks were visualized and analyzed in Cytoscape (version [3.10.3]); clustering was performed with MCL (inflation = 2.0) and enrichment was tested with [BiNGO/stringApp], applying Benjamini–Hochberg correction (FDR < 0.05) (Szklarczyk et al. 2023; Franz et al. 2023).

#### Accession numbers

The Whole Genome Shotgun project has been submitted to the NCBI database under the BioProject number PRJNA1142257, and the genome accession number JBGMDT000000000. The strain has also been deposited in the Culture Collection Ain Shams University with the strain number CCASU-2024–79. The sequence of the *agp* gene was submitted to the NCBI GenBank database with the accession number PX236720.

## Results

### Mz49 isolation and identification

The traditional microbiological identification of Mz49 aligns with characteristics of the *E. cloacae*. Gram staining reveals short, Gram-negative rods. The bacterium is oxidase-negative and, when cultured on MacConkey agar, produces lactose-fermenting colonies. Growth on TSI agar typically shows an alkaline slant with an acidic butt, indicating glucose fermentation. It tested negative indole and positive for urease and citrate utilization (Macfaddin, 2000; Brown et al. 2015). Subsequently, their ability to stimulate plant growth was then evaluated by functional validation.

### Phenotypic characterization of PGPR traits

Mz49 exhibited multiple plant growth-promoting traits under in vitro conditions. The strain produced IAA, demonstrated antioxidant activity, and exhibited anti-inflammatory potential, all of which are linked to enhanced plant growth and stress inhibition. Quantitative analysis revealed potent IAA production, while antioxidant and anti-inflammatory assays showed significant free-radical scavenging activity and erythrocyte membrane stabilization, respectively.

### Determination of IAA production

Salkowski’s reagent (Glickmann and Dessaux 1995; Amer et al. 2021) was used to quantify IAA production by the Mz49 rhizobacterial isolate. A positive pink color reaction was observed, and the mean IAA concentration was 64.89 ± 6.40 µg/mL, as determined from the standard curve.

### Antioxidant and membrane stabilization activity of Mz49

**Fig. 1:**
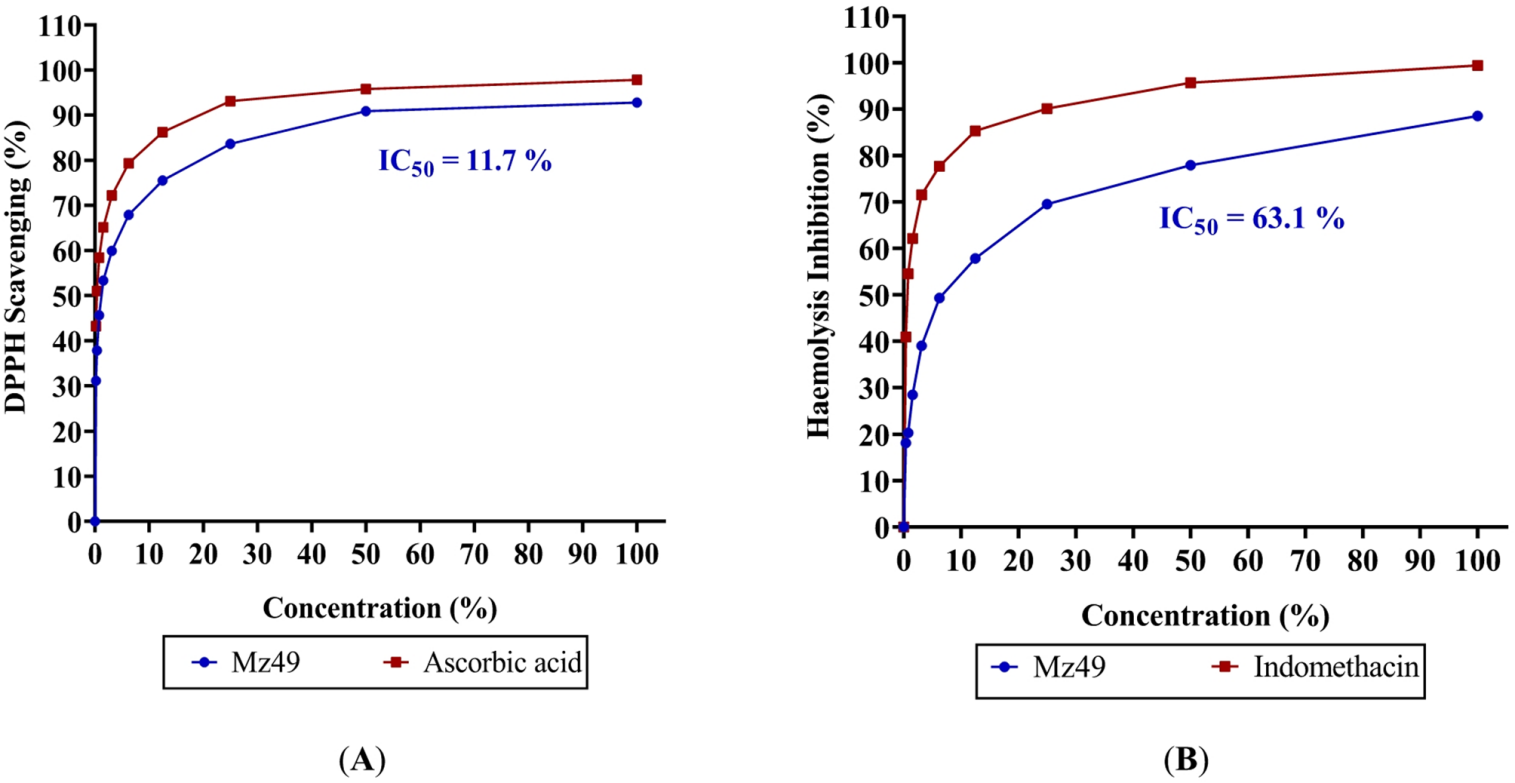
Antioxidant and membrane-stabilizing activities of Mz49. **A** DPPH radical scavenging assay of Mz49 CFS at various concentrations compared with ascorbic acid as the standard. **B** Membrane stabilization (anti-inflammatory) assay of Mz49 CFS at various concentrations compared with indomethacin as the standard

The antioxidant activity of Mz49 was evaluated using the DPPH radical scavenging assay and compared with ascorbic acid as a standard. The CFS of Mz49 exhibited potent, dose-dependent scavenging activity (Fig. 1A), reaching a maximum of 92.8% at undiluted CFS (100% concentration) and decreasing to 31.1% at 0.2% concentration, with an IC₅₀ value of 11.71%. This activity was comparable to that of ascorbic acid, which showed higher potency, achieving 97.8% scavenging at undiluted concentration and retaining 43.2% activity at 0.2%, with a lower IC₅₀ value of 3.15% (Fig. 1A).

The membrane stabilization assay of Mz49 CFS revealed its ability to inhibit hemolysis under hypotonic stress, with an IC₅₀ value of 63.1% (Fig. 1B). At undiluted CFS (100% concentration), Mz49 achieved 88.5% hemolysis inhibition, which decreased progressively with lower concentrations, 77.9% at 50%, 57.8% at 12.5%, and 18.1% at 0.4%. These findings indicate that Mz49 exhibits moderate membrane-stabilizing activity, although it was less effective than the standard drug indomethacin (Fig. 1B).

### Genome assembly and annotation features

**Fig. 2:**
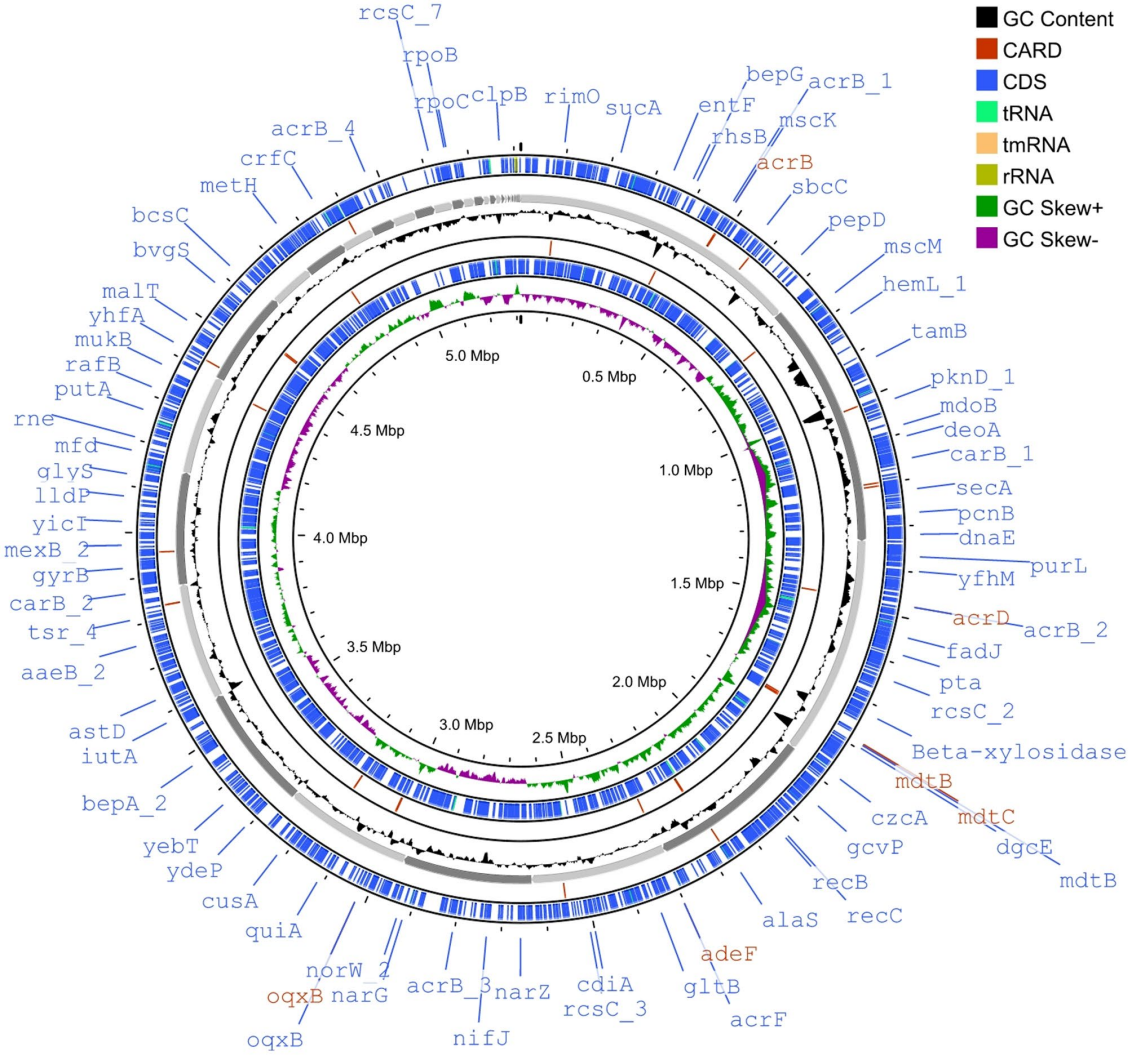
Circular genome map of Mz49. The bacterial chromosome is 5.3 Mb in size. From the outer circle to the inner circle: GC content is plotted in the genome in black color, CARD annotation is shown in red color, coding sequences (CDS) are shown in blue, tRNA operons, tmRNA, and rRNA, and the inner circle indicates the GC skew [(G–C)/(G + C)] positive (green) and negative (purple). A number of interesting genes are highlighted

The assembled draft genome of Mz49 comprised 77 scaffolds with a total length of 5,315,358 bp, an N_50_ of 321,950 bp, and an L_50_ of 6. The DNA GC content is 54.77%, falling within the range for *Enterobacter* species. The genome annotation of Mz49 revealed a total of 5,158 genes. It contained RNA genes comprising 4 rRNA genes and 78 tRNA genes. Mz49 carried one CRISPR array, with two spacers. Assembly and annotation features of Mz49 are provided in Supplementary Table. 1. Of the predicted protein products, 794 were hypothetical proteins, 353 had subsystem assignments, and 4364 had functional assignments. This included 934 proteins with pathway assignments. The circular genome of Mz49 is shown in Fig. 2. The genome includes several specific genes, such as 120 virulence factors (Victors, PATRIC_VF), 64 antibiotic resistance genes (CARD, PATRIC, NDARO), 575 transporter genes (TCDB), and 309 drug target genes (DrugBank, TTD), all identified using their respective databases.

### Phylogenomic features and genome-based taxonomy

**Fig. 3:**
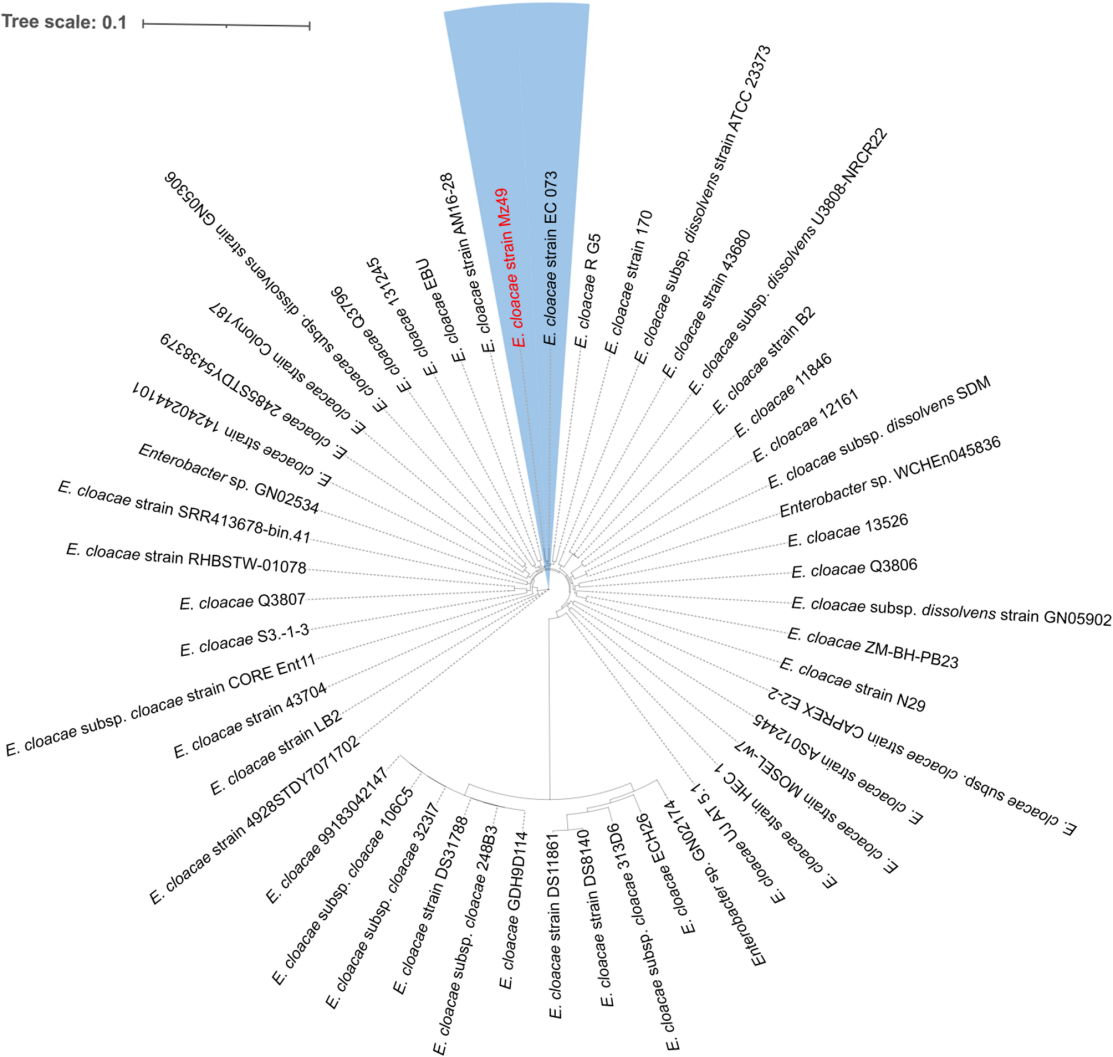
Whole genome phylogenetic tree showing Mz49 sequenced in the current study compared to the closely related genomes. The label of Mz49 is written in red, and the entire clade is highlighted in blue. The figure was created using the iTOL online tool v6.7 (https://itol.embl.de/)

Species assignment of Mz49 was confirmed by calculating the ANIs and the dDDH values in comparison to the most closely related *E. cloacae* strains (identified via BLASTn) in the NCBI database. ANI and dDDH values are shown in Supplementary Table 2. To further elucidate the phylogenomic placement of the Mz49 strain, the draft genome was compared to the genomes of available in the TYGS database. The generated phylogenomic tree, illustrated in Supplementary Fig. 1, showed that Mz49 was clustered with the *E. cloacae* subsp. *dissolvens* ATCC 23373^T^ (based on 70% dDDH threshold). MLST analysis of Mz49 genome showed that it belongs to ST 2056. Additionally, phylogenetic tree depicting the relationship between Mz49 and closely related genomes, retrieved from the BV-BRC database, is shown in Fig. 3. The metadata of the strains included in the phylogenomic analysis are listed in Supplementary Table. 3.

### Comparative genomic analysis and genome alignment

The genome of the *E. cloacae* subsp. *dissolvens* ATCC 23373^T^, a closely related strain, was used as a reference for preliminary comparative analysis based on 16S rRNA sequences. The ordered genome assemblies of Mz49 and *E. cloacae* subsp. *dissolvens* ATCC 23373^T^ (GenBank accession no. AGSY00000000) were analyzed using the Progressive Mauve algorithm via BV-BRC genome alignment tool. Genomic alignment revealed 26 collinear blocks along with several inversion and rearrangement sites (Supplementary Fig. 2). The alignment showed large regions of high similarity across most portions of the two genomes, suggesting that the chromosome structures of both strains are largely conserved. However, the chromosomal region spanning contigs exhibited an inverted orientation, indicating differences in their synteny.

### Functional analysis of the predicted protein products

The Mz49 genome encompasses various functional superclasses, including cellular processes, metabolism, energy production, DNA, RNA, and protein processing, membrane transport, defense mechanisms, stress responses, virulence, cell envelope formation, and cell signaling. Functional annotation using SEED and KEGG pathways identified genes associated with cellular processes, signaling, regulation, and metabolic pathways. The RAST tool predicted that 2037 proteins in the Mz49 genome are assigned to 353 SEED subsystems, which categorize CDSs based on functional relationships. These subsystems, organized within the SEED database, provide insights into the genome's functional roles and metabolic capabilities, with subsystem coverage and distribution illustrating the frequency of genes involved in various biological processes (Supplementary Fig.  3).

The functional analysis was further performed by using the GhostKOALA in the KEGG database revealing the presence of several complete metabolic modules (100 modules) (572 genes) in Mz49 (Table 1A). These modules encompass key metabolic pathways, including carbohydrate metabolism (such as glycolysis and the tricarboxylic acid cycle), amino acid, lipid, nucleotide, glycan, energy, and co-factors and vitamins metabolism. Additionally, pathways for terpenoid and polyketides biosynthesis, as well as secondary metabolite production, were identified, reflecting the organism's metabolic versatility. Signature modules of nitrate assimilation and sulfate-sulfur assimilation were identified. The detection of complete modules suggests a well-integrated metabolic network capable of supporting growth, adaptation, and survival in diverse environments. All complete pathway modules identified in Mz49 are listed in Supplementary Table. 4. To further understand the genomic features of Mz49, functional analysis by COGs was performed (Table 1B), (Supplementary Table. 5). The number of genes assigned to different COGs was determined. 4556 out of the 5158 predicted CDS (88.3%) were assigned to a COG category. This result revealed the main functional gene classes: Carbohydrate transport and metabolism (G), energy production and conversion (C), amino acid transport and metabolism (E) and transcription (K), representing 29.15% of the predicted CDS assigned to COG categories. Another high percentage cluster (20.7%) represented genes involved in cell wall/membrane/envelope biogenesis (M), Translation, ribosomal structure and biogenesis (J), Coenzyme transport and metabolism (H) and signal transduction (T). Almost 21.84% of the predicted CDS are poorly characterized with function unknown (S).

### Predicted secondary metabolite BGCs

The potential secondary metabolites produced by Mz49 were predicted by the antiSMASH server. Four potential secondary metabolite-encoding BGCs identified in the genome of Mz49 are shown in Table 2 and Supplementary Fig. 4. Mz49 genome included gene clusters for the production of NI-siderophore (aerobactin), NRP-metallophore, NRPS (enterobactin), arylpolyene, NRPS, Type I Polyketide Synthases (T1PKS), and thiopeptide (O-antigen). A gene cluster with NRP-metallophore, NRPS, T1PKS biosynthesis domains was found (Supplementary Fig. 4b), and it showed a 100% similarity to a known enterobactin BGC. Strain Mz49 additionally had a gene cluster NI-siderophore (Supplementary Fig. 4a) showing 66% similarity to aerobactin BGC. Two additional BGCs were identified, corresponding to NRPS and terpene clusters (Supplementary Fig. 4c and 4d respectively. They showed 88% and 14% similarity to arylpolyene and O-antigen BGC, respectively. Another gene cluster containing NRPS and T1PKS elements (Supplementary Fig. 4e) was observed, showing no similarity to known BGCs. Furthermore, each identified BGC obtained from anti- SMASH was compared against the NCBI database using BlastP (protein–protein blast). Mz49 BGCs were found to be conserved among *E. cloacae* species.

**Table 2:** Secondary metabolite BGCs predicted in the genome of Mz49

Isolate	Region (Contig, cluster)	Type	From	To	Most similar known cluster	Similarity	Core biosynthetic genes	Additional bio-synthetic genes	
Mz49	a (1.1)	NI-siderophore	361,723	394,151	aerobactin	Other	66%	2	3
	b (3.1)	NRP-metallophore,NRPS	367,082	421,221	enterobactin	NRP	100%	3	12
	c (8.1)	Arylpolyene	74,737	118,330	arylpolyene	Other	88%	2	13
	d (9.1)	NRPS,T1PKS	154,458	202,194	–	–	–	1	4
	e (19.1)	Thiopeptide	1	19,318	O-antigen	Saccharide	14%	2	1

## Plant-related genes

Mz49 contained 1840 genes that are linked to plant growth-promotion, directly by biofertilization, phytohormone production, and bioremediation, or indirectly by stress control, promoting plant colonization, and immune stimulation (Supplementary Fig. 5; Supplementary Table. 6 and 7).

### Biofertilization and nutrient acquisition pathways

The genome of Mz49 encodes genes involved in carbon dioxide fixation, iron and nitrogen acquisition, sulfur assimilation, and phosphate and potassium solubilization, underscoring its capacity to enhance nutrient availability in the rhizosphere.

Mz49 carried CO₂ fixation-related genes *ppc, icd, cooF, porEF, cah, cynT, can, nifJ, por,* and the transcriptional regulators *cbbR, cmpR,* and *ndhR*, supporting photosynthesis. Nitrogen acquisition and fixation by Mz49 are supported by a wide set of functional genes encompassing multiple pathways: allantoin utilization (*allC, allR, hpxB*), ammonium assimilation and metabolism (*nitABR, gdhA, glnABDEGHK, gltBD, ntrBC*), atmospheric nitrogen fixation (*hyaD/hybD/hupD, hyfABCGHI, hypABCDEF, nifFJMSU*), denitrification (*narGHIJKX, nasACDEF, nirBD, norRVW, nosX, fnr, narBLPQX, yfdC*), hydroxylamine usage (*hcp*), and urea utilization (*ureABCDEFGJ, urtABCDE, atzF, uca/dur/urd, ycgI*). These systems are further regulated by nitrogen acquisition regulators including *fdnGHI, frdABCD, glnBD, ntrABC, modE, narLPQX, exoR, acoR, draG, fixJ,* and *glnKZ*.

Sulfur assimilation is likewise supported by genes involved in alkanesulfonate transport and degradation (*ssuBCDE*), DMSO reduction (*dmsABCD*), sulfate transport and reduction (*cysACDHIJKMNPTWZ*), taurine utilization (*tauABCD, ggt*), thionate degradation (*ttrR*), and thiosulfate degradation (*glpE*). Together, these systems highlight the strong potential of Mz49 to mobilize essential nutrients and enhance soil fertility in the rhizosphere.

**Fig. 4:**
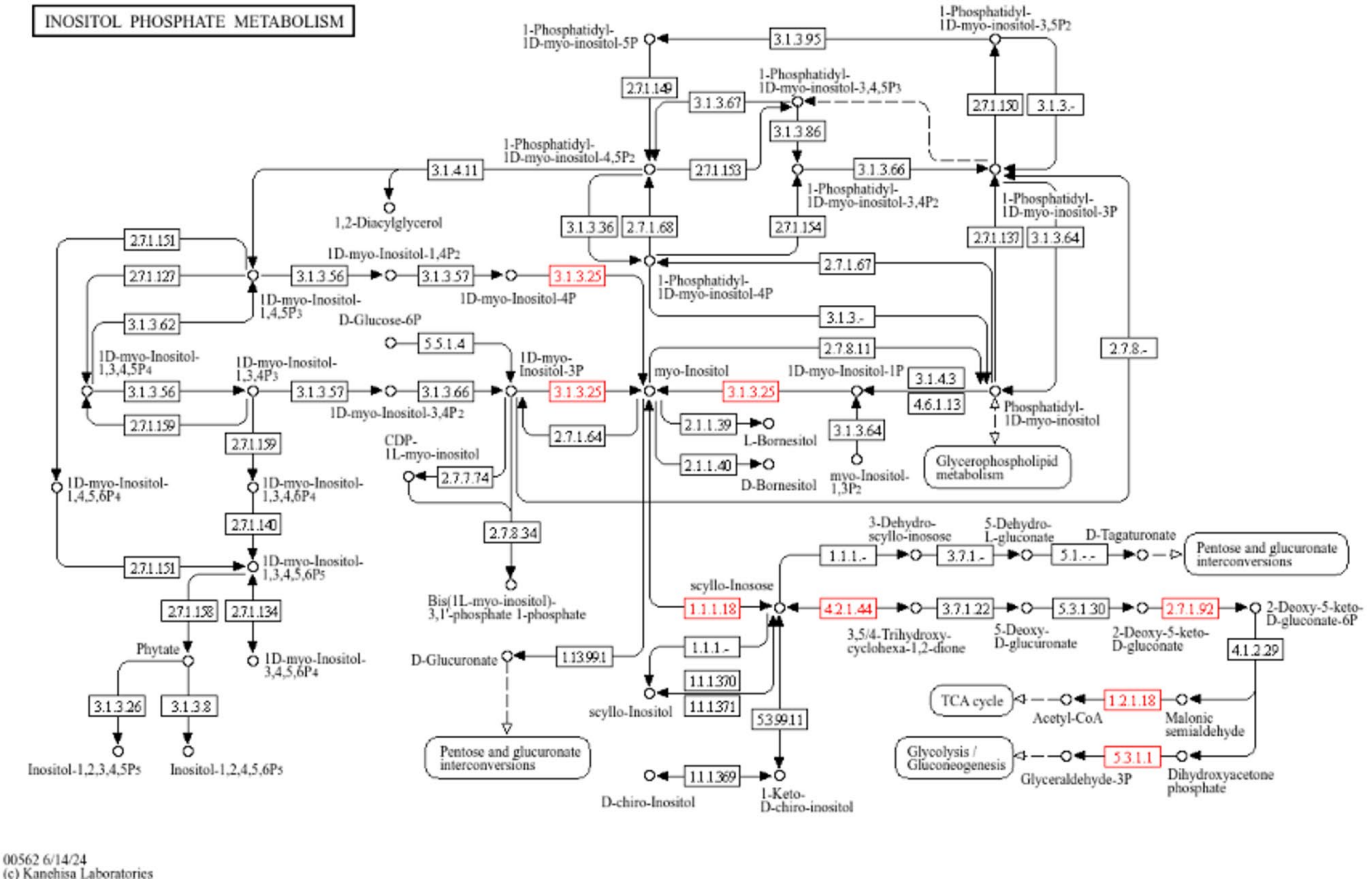
KEGG pathway mapping of inositol phosphate metabolism in strain Mz49, highlighting annotated genes (in red) involved in phytic acid degradation. Detected enzymes include myo-inositol 2-dehydrogenase 1 (EC 1.1.1.18), malonate-semialdehyde dehydrogenase [inositol] (EC 1.2.1.18), 5-keto-2-deoxygluconokinase (EC 2.7.1.92), inositol-1-monophosphatase *suhB* (EC 3.1.3.25), 2-hydroxy-6-oxo-6-phenylhexa-2, 4-dienoate hydrolase (EC 3.7.1), inosose dehydratase (EC 4.2.1.44), and triosephosphate isomerase (EC 5.3.1.1)

**Fig. 5:**
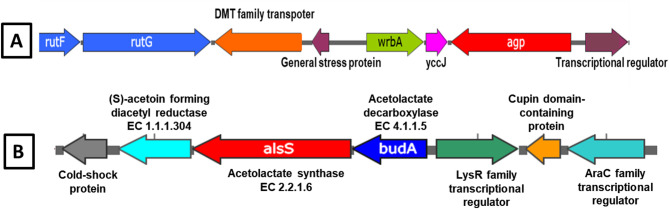
Genetic organization of **A** the phytase (*agp*) gene cluster and **B** the acetoin/2, 3-butanediol biosynthesis operon in strain Mz49. **A** The red arrow indicates the *agp* gene (EC 3.1.3.10), encoding phytase enzyme involved in phytate degradation. The pink arrow denotes *yccJ*, a regulator associated with stress survival and pathogenicity. The green arrow highlights *wrbA*, which converts 6-hydroxy-2, 4-dihydroxyquinoline (HDHQ) to PQQ, a redox cofactor. The orange arrow marks a DMT family transporter protein contributing to metal resistance, homeostasis, and divalent metal uptake. Blue arrows represent *rut* family genes, which enable pyrimidine utilization under nitrogen- and carbon-limiting conditions. **B** The red arrows denote acetolactate synthase (*als*, EC 2.2.1.6) and acetolactate decarboxylase (*budA*, EC 4.1.1.5), which sequentially convert pyruvate to α-acetolactate and then to acetoin. Neighboring genes (various colors) encode predicted regulatory and accessory proteins

Phosphate and potassium solubilization in strain Mz49 are mediated by the production of organic acids (e.g., acetic, propionic, butyric, gluconic, and lactic acids) as well as inorganic acids (carbonic, nitric, and sulfuric acids), in addition to phosphonate transport and degradation pathways. Enzymatic phosphate solubilization is supported by the presence of genes encoding acid phosphatase (*aphA*), alkaline phosphatase (*phoA*), inorganic phosphatase (*ppa*), and exopolyphosphatase (*gppA*). Phytate degradation, which involves the hydrolysis of phytic acid (myo-inositol hexakisphosphate), a major organic phosphorus reserve in plant tissues, into inositol and inorganic phosphate, further enhances phosphorus bioavailability. Genomic analysis of Mz49 revealed the presence of key phytase-related genes, including *agp* (EC 3.1.3.10), a bifunctional glucose-1-phosphatase/inositol phosphatase encoding phytase enzymes responsible for hydrolyzing phytic acid under acidic conditions, and *suhB* (EC 3.1.3.25), an inositol-1-monophosphatase commonly linked to phytate hydrolysis. The annotated genes related to inositol phosphate metabolism, as detected by KEGG pathways, are illustrated in Fig. 4, and a genetic map of the *agp* locus with its neighboring genes is presented in Fig. 5A.

### Production of Indole-3-acetic acid and other phytohormones

In Mz49, the production of IAA is likely facilitated by a combination of tryptophan-dependent pathways, with several key genes potentially contributing to this biosynthesis. Genes such as *iaaT*, *yedL*, and *ysnE* might be involved in the transport or conversion of tryptophan or related intermediates to IAA via the indole-3-pyruvate (IPA) or indole-3-acetamide (IAM) pathways. The *ipdC* and *ppdC* genes are associated with the conversion of tryptophan into IAA through the IPA key pathway. Additionally, the *amiE* gene, which is linked to the IAM pathway, could contribute to IAA biosynthesis by hydrolyzing IAM into IAA. Genes involved in tryptophan biosynthesis, such as *trpABC*, likely supply the precursor tryptophan necessary for these pathways, while genes like *nitA*, *nitB*, and *nitR* might play a regulatory role in nitrogen metabolism, indirectly supporting IAA production.

Other genes potentially involved in various plant hormone pathways, particularly those regulating growth and stress response have been identified in the genome of Mz49. Genes such as *miaA*, *ipt* are likely involved in the biosynthesis of cytokinins, promoting cell division and growth, while *miaB* and *miaE* may participate in cytokinin metabolism. *puuAB*, and related genes involved in putrescine degradation could influence polyamine levels, which in turn regulate auxin transport and plant development. The *patA1* gene suggests a role in polyamine metabolism, potentially impacting auxin and cytokinin homeostasis. Additionally, genes like *fadA*, and *fadI* involved in fatty acid degradation could influence jasmonic acid synthesis, a key hormone in stress responses. Other genes such as *gpt* and *ndmA* may be involved in xanthine metabolism, cellular transport, and stress-related mechanisms, indirectly affecting hormonal regulation (Supplementary Table. 6).

Acetoin and 2,3-butanediol are volatile organic compounds (VOCs) emitted by many PGPB to enhance plant growth. The main pathway for the production of these VOCs by Mz49, is via the sequential action of enzymes coded by the operon *budABC* detected in its genome. A genetic map of the *budA* locus and its neighboring genes is shown in Fig. 5B.

### Bioremediation and heavy metal detoxification

Strain Mz49 carries a diverse repertoire of genes that contribute to soil health through xenobiotic degradation (*atzF, dmpB,* and *xylA*) and resistance to a wide range of heavy metals, including antimony, arsenic, cadmium, cobalt, copper, iron, manganese, mercury, nickel, selenium, silver, tellurium, and zinc (Supplementary Table. 7). These genes encode efflux pumps, transporters, reductases, and regulatory proteins that collectively maintain metal ion homeostasis and protect against toxicity. Key systems include the *ars* operon and PST-Pho-Pit transporters for antimony/arsenic, *czcD*, *zntA*, and *cus* family transporters for cadmium, copper, and silver, *mnt* and *sit/yfe* systems for manganese and iron, and *znuABC* and *zntABR* for zinc. Additional mechanisms involve *exoR*-regulated mercury resistance, *ddp* and *rcnA*-mediated nickel resistance, *selABDU*/ynfEH genes for selenium, and *teh*/*ter* genes for tellurium, alongside broad regulators such as *cutA*, *cusR*, *copR*, *silR*, and the TonB-ExbB-ExbD complex.

### Stress tolerance mechanisms

Genomic features linked to abiotic stress resistance, including oxidative stress, temperature stress, osmotic and salinity stress, and herbicidal stress, were identified in Mz49 (Supplementary Table. 7).

The genome of Mz49 harbors a comprehensive suite of enzymes and regulatory elements that facilitate oxidative stress management, thereby enhancing bacterial survival across diverse environments. Key antioxidant enzymes include superoxide dismutases (*sodABC*), catalases (*katE*, *katN*), and peroxide detoxifiers such as *ahpC, ahpF, bcp, cpo, efeB, grxA,* and *gor*. The hydrogen peroxide sensor gene *oxyR* regulates multiple oxidative stress-related genes, including glutathione reductase (*katG, gor*), *ahpC*, *ahpF*, *dpsA* (DNA protection during starvation), *fur* (ferric uptake regulator), and *grxA* (glutaredoxin). Additional protective mechanisms consist of glutathione S-transferases (*gst*), a glutathione ABC transporter (*gsiABCD*), glutathione peroxidases (*btuE*), and γ-glutamyl transpeptidase (*ggt*). The genome also encodes genes for sulfur-containing compound synthesis via the H_2_S volatile pathway (*cysABCDHIJMNPQSTWZ*), contributing to oxidative defense. Genes involved in niacin biosynthesis (*deoD, iunH, mazG, nadABCDERX, nudC, pncBC, punA, surE, ushA, yjjG,* and *yrfG*) support NAD(P)H production, essential for redox reactions neutralizing reactive oxygen species. Furthermore, nitric oxide reductase genes (*norRVW* and *hmp*), regulatory systems (*arcAB, ompCFR, oxyR,* and *soxSR*), and chaperones/oxidoreductases (*grxACD*) collectively maintain cellular homeostasis under oxidative stress (Supplementary Table. 7).

The Mz49 genome harbors a set of genes that contribute to temperature stress tolerance, enabling adaptation to both high and low-temperature conditions. For high-temperature tolerance, genes involved in heat shock response (*clpABPXC, djlA, dks, dnaJK, groEL, groES, htpX, ibpAB*) encode molecular chaperones that prevent protein misfolding and aggregation. Cold shock proteins (*cspA*) maintain RNA stability and efficient translation; several low-temperature-related enzymes (*ariR, gdh, hutD, ydaE, yfhD, yfhF*) facilitate metabolic adjustments necessary for survival in cold environments.

The Mz49 genome encodes several mechanisms for surviving acidic stress. The Evg-Emr system, regulated by *evgA* and *evgS*, activates stress responses crucial for acid resistance. The *mdcF* gene aids in oxalate-induced acid tolerance. Polyamine metabolism, regulated by various genes, helps buffer intracellular pH. Additionally, genes like *phoP*, *phoQ*, *rpoS*, and *yciG* coordinate stress responses, boosting acid resistance.

The genome of Mz49 contains genes essential for osmotic and salinity stress adaptation, including *betaA*/B homologs for glycine betaine metabolism and *mtlD* for mannitol biosynthesis. It encodes glutamate transporters (*gltIJKL*) and mechanosensitive ion channels (*mscKLM, mscKLMS,* and *ykuT*) that mediate osmotic pressure responses. Osmoprotectant synthesis and transport genes include proline-related (*argABCDEHO, gbuABC,* and *proABCY*), trehalose-related (*glvA, malK, otsAB,* and *treABCRSXYZ*), and ectoine-related (*doeBX, ectB,* and *lysC*) pathways. Ion homeostasis is regulated by Na + /H + antiporters (*nhaABKR, yrbG, araE,* and *rfnABCDEFG*), K + transport systems (*kdpABCDEFG, sapABCF, trkBD, kdpA–F,* and *trkA–G*), and Mg2 + transporters (*corAC, mgtE, phoPQ, pmrAB,* and *yhdT*). Polyamine metabolism and transport genes (*ydcTUVS, gsp, ldcC/cadA, paiA,* and *potA–I*) control spermidine and putrescine levels. Additional regulatory genes (*cvrA, kefA–G, sapA–F, emrB, osmB,* and *rhlE*) support survival under stress, while ATPase genes (*atpABCDEFGHI*) help maintain energy and ion gradients. DNA protection and stress response are facilitated by *dps*, Lon protease, and SpoT.

The Mz49 genome encodes multiple mechanisms to neutralize herbicidal stress. It includes genes for the degradation of chloroacetanilide (*fdx, hca, cndBC*), organophosphates (*glpABD, opaA, pepQP*), and paraquat (*aldH, dhaS, clsA_B, ybhO, ywiE, csbC, pqiB, yfkH, yfkJ, wzb, yflA*). Additionally, genes involved in toxoflavin metabolism (*ribAD, toxFI, oprM, emhC, ttgC, cusC, adeK, smeF, mtrE, cmeC, gesC*) contribute to the detoxification processes.

### Genes enhancing plant colonization and immune stimulation

The genome of Mz49 harbors genes associated with exopolysaccharide production, adhesion, motility, as well as additional factors that support colonization and long-term plant association (Supplementary Table. 7).

Motility is a key trait for soil-rhizobacteria, facilitating their movement and colonization of plant roots. Several genes related to chemotaxis and flagella biosynthesis/assembly were identified in the Mz49 genome (Supplementary Table. 7). Annotated gene clusters associated with motility and host attachment included *fliABCDEFGHIJKLMNOPQRSTYZ*, *secABDEFGMY*, *mglABC*, *rbsABCDKR*, *cheABRVWYZ*, *lapABCEF*, *motABD*, *pilKTM*, *rfbABC*, *oprDFM*, and *epsABCDEFGHIJKLM*. The genome of Mz49 encodes the complete set of genes for cellulose biosynthesis (*bcsABCZ*) and curli fiber production (*csgABCDEFG*), which are commonly co-expressed. It also carries genes for colanic acid biosynthesis and translocation (*wcaABCDEFHIJKLM*), an exopolysaccharide essential for biofilm formation in *Enterobacteriaceae*. In addition, the *srfABCD* operon, associated with root adhesion, was identified.

The genome of Mz49 encodes *phzF* and *ubiC*, which are involved in the biosynthesis of phenazine and 4-hydroxybenzoate, compounds with antibacterial activity against plant pathogens. Several genes linked to chitinolytic activity were also identified, including *cbp, chpG, bcsZ, chiA, nagZ*, as well as genes encoding chitin acetylase and putative chitinase; such enzymes are effective against insects and fungi and have been reported in PGPR from *Enterobacter*, *Klebsiella*, *Pantoea*, and *Serratia*. In addition, genes related to fungicidal metabolism (*asm, tktAB, ctdC, ribAD,* and *toxIF*) and insecticidal activity (*patD, puuED,* and *tccC*) were annotated.

### Pathogenicity prediction and the identification of ARGs and virulence genes

PathogenFinder predicted that Mz49 has a mean probability of 0.803 of being pathogenic to humans, with matches to eight pathogenic families. Genome analysis revealed the presence of ARGs, including *bla*_CMH-3_ (conferring resistance to cephalosporins) and *fosA2* (conferring resistance to fosfomycin). In addition, several efflux pump genes (*emrB*, *kpnEF*, *acrA*, *msbA*, and *oqxA*) were identified, providing resistance across multiple antibiotic classes. Analysis of mobile genetic elements detected two plasmid replicons, IncFIB(pECLA) and IncFII(pECLA); however, neither carried antibiotic resistance genes.

Virulome analysis of the Mz49 genome revealed the presence of several putative virulence genes spanning multiple functional categories. These included *acrA* and *acrB* (acriflavine resistance proteins), *csgG* (curli production assembly/transport protein), *entB* (isochorismatase), *fepB* (iron-enterobactin transporter membrane protein), *fepG* (iron-enterobactin transporter permease), *galF* (UTP-glucose-1-phosphate uridylyltransferase subunit), *gnd* (6-phosphogluconate dehydrogenase), *ompA* (outer membrane protein A), and *rcsB* (transcriptional regulator).

### Metabolic profile of Mz49 CFS

The GC–MS analysis was utilized to identify the compounds present in the CFS of Mz49. The chromatogram displayed distinct peaks, indicating the presence of various compounds (Supplementary Fig. 6). CFS of Mz49 revealed the presence of several bioactive metabolites with potential plant growth-promoting and anti-stress roles (Table 3). The most abundant compound was palmitic acid (20.82%), followed by stearic acid (4.20%) and 2, 3-butanediol (5.03%), a VOC known to induce systemic resistance and enhance drought tolerance in plants. Short- and medium-chain fatty acids such as propanoic acid (1.63%), hexanoic acid (0.89%), octanoic acid (0.63%), and myristic acid (0.69%) were detected, which are associated with antimicrobial activity and improved plant defense. Additionally, tyrosol (1.74%) and azelaic acid (0.86%) were identified, both recognized for their antioxidant properties and roles in plant stress response. Other metabolites included butanedioic acid (succinic acid, 1.39%), involved in cellular respiration and metabolic regulation, and D-pinitol (1.93%), a compound linked to osmoprotection and drought tolerance.

**Table 3:** Major constituents of the CFS of Mz49 identified using GC–MS analysis

Compound no	RT	% Area	Compound name	Molecular formula	Class
A	5.03	5.75	2,3-Butanediol	C_4_H_10_O_2_	Polyols (1,2 diol)
B	6.57	1.63	Propanoic acid	C_3_H_6_O_2_	Short chain fatty acid
C	6.71	0.89	Hexanoic acid (caproic acid)	C_6_H_12_O_2_	Medium chain fatty acid
D	11.48	0.63	Octanoic acid(caprylic acid)	C_8_H_16_O_2_	Medium chain fatty acid
E	12.95	1.39	Butanedioic acid (succinic acid)	C_4_H_6_O_4_	Dicarboxylic acid
F	19	1.74	Tyrosol	C_8_H_10_O_2_	Phenylethanole
G	23.99	0.86	Azelaic acid	C_9_H_16_O_4_	Saturated dicarboxylic acid
H	24.85	0.69	Myristic acid	C_14_H_28_O_2_	Long-chain fatty acid
I	24.92	1.93	D-pinitol	C_7_H_14_O_6_	Cyclic hexanol
J	28.79	20.82	Palmitic acid	C_16_H_32_O_2_	Long-chain fatty acid
K	32.17	4.2	Stearic acid	C_18_H_36_O_2_	Long-chain fatty acid

RT, Retention time

### Predicted features of the phytase enzyme encoded by the *agp* gene

#### Physicochemical properties

Analysis of the phytase enzyme using ExPASy ProtParam showed that it comprised 413 amino acids with a predicted molecular weight of 45 kDa and a theoretical isoelectric point (pI) of 5.76, suggesting a predominantly acidic nature. The aliphatic index (76.30) suggests moderate thermostability, while the negative GRAVY score (− 0.420) indicates a hydrophilic nature, implying favorable interactions with aqueous environments.

#### Conserved domain and motif analysis

**Fig. 6:**
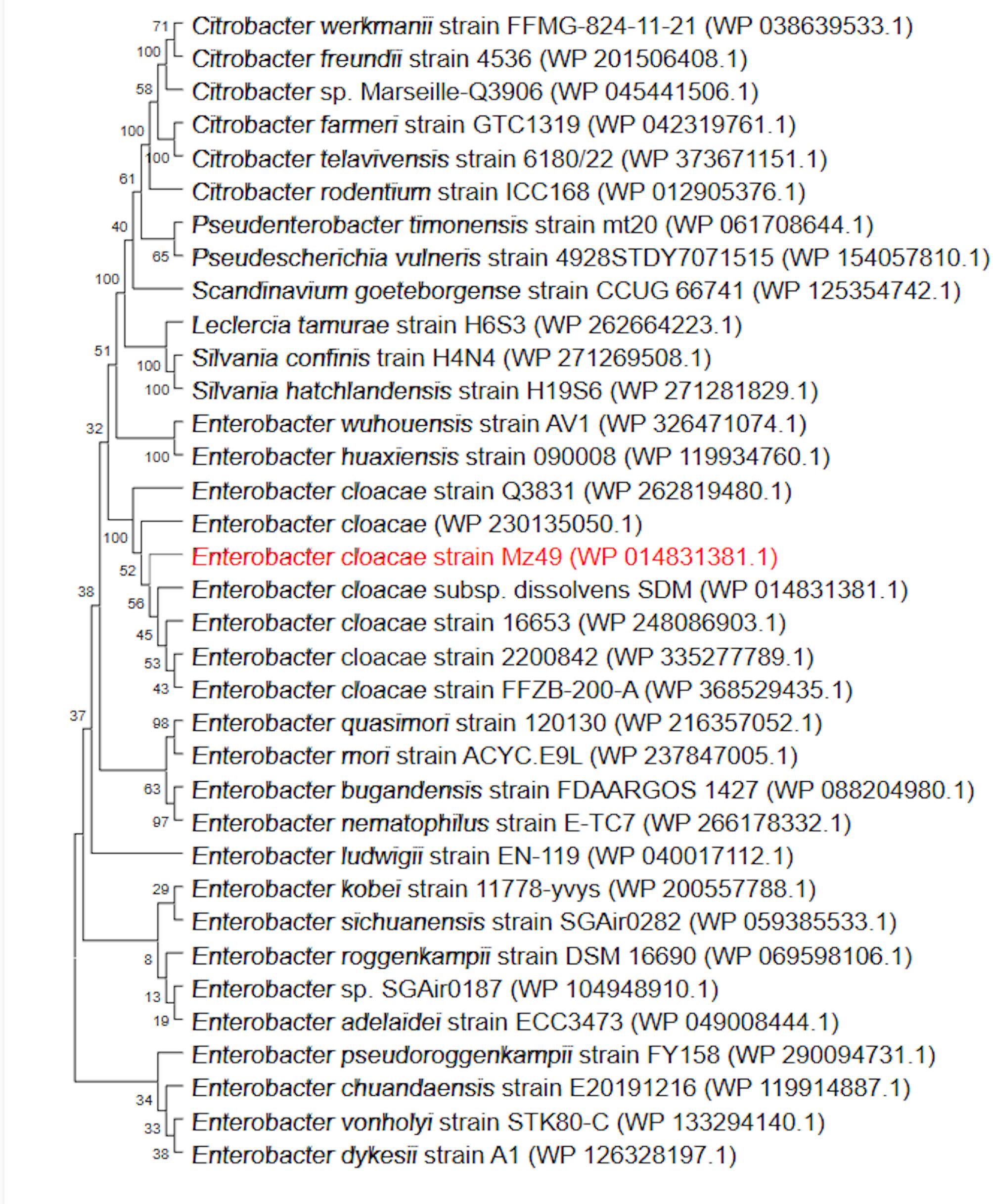
Phylogenetic tree of the *agp*-encoded phytase from strain Mz49 and homologous phytase sequences from *Enterobacteriaceae*, constructed using the neighbor-joining method. GenBank accession numbers are in parentheses. Phylogenetic analysis showed that *agp* is closely related to phytases from closely related genomes and identical to the phytase of *E. cloacae* strain subsp. *dissolvens* SDM (WP_014831381.1)

Domain prediction using InterProScan and Pfam identified a histidine acid phosphatase (HAP) domain, which is characteristic of phytases. Conserved active-site residues were detected, including RH (positions 39–40), *R* (43), *R* (116), and HD (311–312), all of which are highly conserved among known phytases. The histidine acid phosphatase phosphohistidine signature motif *LeqVliMsRHNlRaP* (residues 31–45) was also identified, consistent with [3-phytase/6-phytase] activity (Supplementary Fig. 7). Phylogenetic analysis was conducted by comparing the amino acid sequence of the *agp*-encoded phytase with homologous sequences from other Enterobacteriaceae using the neighbor-joining method. Multiple sequence alignment confirmed strong conservation of catalytic residues and overall domain architecture. The phylogenetic tree positioned the Mz49 phytase in close relation to *E. cloacae* subsp. *dissolvens* SDM (WP_014831381.1), with 100% sequence identity, indicating strong evolutionary conservation (Fig. 6).

#### Secondary and tertiary structure modeling

Phyre2 analysis predicted that the protein consists of approximately 33% α-helices, 11% β-strands, and the remaining 56% random coils and loops. Disorder analysis indicated only ~ 2% disordered regions, suggesting a predominantly structured conformation suitable for stable enzymatic function (Supplementary Fig. 8). Phyre2 generated a high-confidence 3D model using templates from structurally characterized phytases. The model achieved 100% confidence across 94% coverage of the sequence (Supplementary Fig. 9).

#### Protein–protein interaction network

**Fig. 7:**
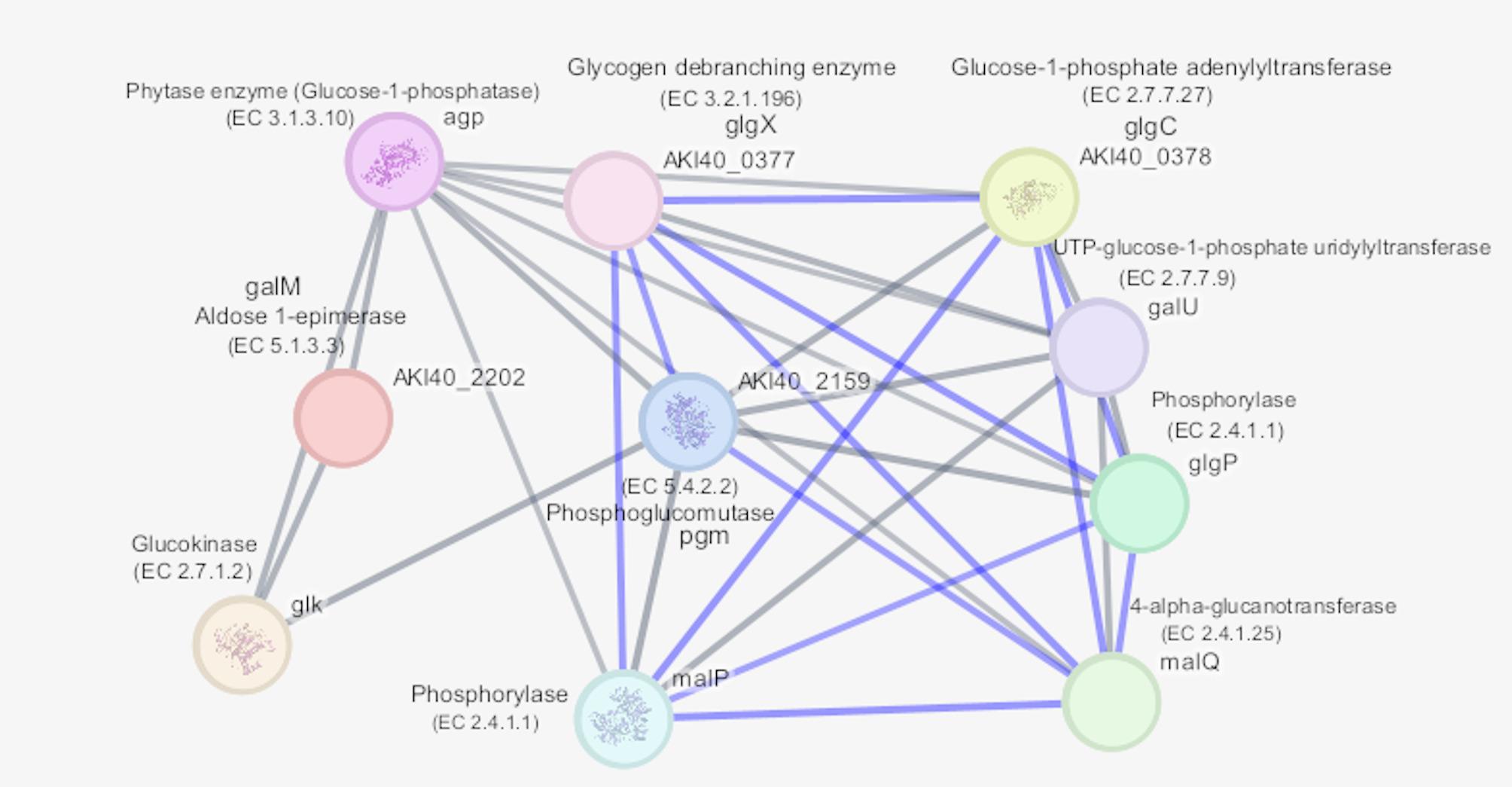
Protein–protein interaction (PPI) network of the *Agp* phytase enzyme and associated proteins in Mz49, generated using Cytoscape (version 3.10.3). The network shows 11 nodes and 43 edges, indicating significant functional associations (PPI enrichment p-value 6.3 × 10⁻^14^). Key interacting proteins include phosphoglucomutase (*pgm*), glucokinase (*glk*), aldose 1-epimerase (*galM*), and glycogen debranching enzyme (*glgX*), all linked to carbohydrate and glycogen metabolism. Edge thickness represents the confidence score of interactions, with darker lines indicating stronger associations. Blue edges respresent co-occurrence

The STRING analysis visualized in Cytoscape, of the *Agp* protein revealed a highly interconnected network of 11 proteins with 43 predicted interactions, showing significant enrichment (PPI enrichment p-value 6.3 × 10⁻^14^). Key associations were observed with phosphoglucomutase (*pgm*; score 0.910), aldose 1-epimerase (*galM*; 0.905), and glucokinase (*glk*; 0.900), highlighting their roles in glycogen and carbohydrate metabolism. Gene Ontology (GO) enrichment analysis indicated functional involvement in alpha-glucan catabolic (*malQ*) processes, glycogen debranching (*glgX*), and phosphorylase (*glgP*) activity (FDR < 0.05). KEGG pathway mapping further supported these findings, showing enrichment in starch and sucrose metabolism, galactose metabolism, and glycolysis/gluconeogenesis pathways. These results suggest that *Agp* and its network partners are closely associated with phosphate-dependent carbohydrate metabolism, implying a functional link between phytase activity and the generation of phosphate required to drive these pathways in Mz49 (Fig. 7).

## Discussion

This study primarily investigates the plant growth-promoting potential of *E. cloacae* isolate Mz49. The results focused on the genomic prediction and in silico protein characterization of the phytase enzyme, supported by functional traits such as IAA production, stress tolerance, and other PGPR-associated features. While comprehensive genomic and metabolomic analysis were conducted, the primary findings related to phosphorus mobilization through phytase activity and the ability to withstand abiotic stresses, require sharper integration. Below, we synthesize the data into a cohesive interpretation, linking genomic evidence, phenotypic assays, and metabolic outputs to practical agricultural benefits.

Rhizobacterium Mz49 was isolated and characterized from maize (*Zea mays* L.), investigated for potential activity to enhance plant growth and resist stress. *E. cloacae* has been found in various environments and maintains a close association with multiple hosts, including plants, water, animals, and humans (Davin-Regli and Pagès, 2015). A comprehensive analysis was done to the strain Mz49 through traditional microbiological methods and advanced genomic and metabolomic techniques. Research has highlighted the environmental and agricultural significance of *E. cloacae* in various crops, including *Curcuma longa* (Alrajeh and Sherif 2024), maize (Xue et al. 2024), and groundnut nodules (Ramakrishnan et al. 2023), largely due to its potential role in promoting plant growth. Whole-genome analysis was done to identify *E. cloacae* genes associated with plant growth promotion, phytohormone production, and stress resistance.

The phenotypic identification of Mz49 aligns closely with conventional microbiological descriptions of this species, the strain was confirmed as a Gram-negative rod capable of fermenting lactose on MacConkey agar and producing an alkaline slant with an acidic butt on TSI agar. Its metabolic profile, with negative indole and positive urease and citrate utilization tests, further confirms its classification as an *E. cloacae* strain (Bergey et al. 1989; Davin-Regli and Pagès, 2015).

Several phenotypic assays were selected to evaluate key plant growth-promoting traits of Mz49. IAA production supports root development and nutrient acquisition, while antioxidant activity indicates the potential to overcome oxidative stress in plants. Anti-inflammatory potential was assessed as an additional trait reflecting the strain’s ability to modulate stress responses and promote overall plant health in the rhizosphere (Khadem et al. 2014; Shrivastava et al. 2023; Sun et al. 2024; Salem et al. 2024).

The ability to withstand abiotic stress is critical for a PGPR strain intended for field application. The antioxidant activity of the CFS of Mz49, was assessed through the DPPH radical scavenging assay, demonstrated significant potential with a maximum activity of 92.8% at undiluted CFS and an IC_50_ value of 11.71%. While slightly lower than the standard ascorbic acid (97.8%, IC_50_ = 3.15%), this result is consistent with previous studies, which report the production of antioxidant compounds in plant-associated bacteria (Vincze et al. 2024). The observed dose-dependent activity suggests the strain produces bioactive metabolites that neutralize free radicals. Similar antioxidant capabilities have been attributed to bacterial production of enzymes like catalase and superoxide dismutase, as well as secondary metabolites like phenolics and flavonoids (Prakash Shyam et al. 2021). The membrane stabilization assay demonstrated that the CFS of Mz49 effectively inhibits hemolysis under hypotonic stress, with an IC_50_ value of 63.1%. The inhibitory effect was concentration-dependent, with the highest protection (88.5%) observed at undiluted CFS (100%), which progressively decreased at lower concentrations. This suggests that Mz49 possesses moderate membrane-stabilizing properties, compared to the standard indomethacin. Membrane stabilization is a critical parameter in evaluating anti-inflammatory potential. The observed hemolysis inhibition by Mz49 suggests the presence of bioactive metabolites capable of stabilizing erythrocyte membranes, aligning with previous findings on bacterial-derived compounds with anti-inflammatory properties (Rizvi et al. 2022; Chen et al. 2024; Tanwy et al. 2024). Similar membrane-stabilizing effects have been reported in bacterial metabolites, such as exopolysaccharides and biosurfactants, which interact with membrane lipids to enhance stability under stress conditions (Santos et al. 2016).

Mz49 exhibits multiple genomic adaptations that collectively enhance survival under drought, salinity, and oxidative stress conditions that often limit crop productivity. Genes encoding polyamine transport and metabolism systems (e.g., *potABCD*, *ydcTUVS*) enable the uptake and turnover of spermidine and putrescine, compounds essential for maintaining cellular integrity under osmotic stress. Complementing this, mechanosensitive ion channel genes (*mscK*, *mscL*) and potassium transport operons (*kdp*, *trk*) likely help the bacterium regulate pressure in fluctuating soil moisture conditions. The presence of nitric oxide reductases (*norRVW*) and regulatory elements such as *oxyR* and *soxSR* suggests multiple mechanisms to neutralize reactive oxygen species generated during plant–microbe interactions and under drought stress. These genomic insights align with phenotypic assays showing Mz49’s persistence in the rhizosphere which also indirectly benefit plants by stabilizing the microbiome during stress, reducing the likelihood of pathogen overgrowth. The genome of Mz49 encodes genes enabling adaptation to multiple environmental stresses. Oxidative stress is countered by catalases, superoxide dismutases, glutathione enzymes, and regulatory elements like *oxyR* (Brill et al. 2011). Osmotic stress tolerance is supported by osmoprotectant synthesis (glycine betaine, proline, mannitol), polyamine metabolism, potassium transport, and mechanosensitive channels. Temperature extremes are managed via heat shock proteins for high-temperature tolerance and cold shock proteins plus metabolic enzymes for low-temperature survival. Together, these genetic traits allow Mz49 to endure oxidative, osmotic, and temperature stresses, promoting persistence in diverse environments. Overexpression of catalase genes in *E. coli* has been shown to enhance salt stress tolerance in transgenic indica rice (Moriwaki et al. 2008) and increase salt resistance in jute plants (Islam et al. 2013). In strain Mz49, the presence of *betB*, a gene involved in the glycine betaine production pathway and known for its osmoprotective effects, aligns with previous findings in *Bacillus subtilis* (Wani et al. 2016). The identification of genes like *proABCY*, which are involved in proline synthesis, suggests a mechanism for drought stress mitigation, as proline production is a well-known strategy exhibited by beneficial microbes to counter drought conditions. Mz49 genome harbors a lot of genes that contribute to temperature stress tolerance, enabling adaptation to both high and low-temperature conditions. These genetic components collectively provide Mz49 with the ability to endure and thrive under extreme temperature fluctuations, ensuring its persistence in diverse ecological conditions (Keto-Timonen et al. 2016; Cardoza and Singh 2024; Alam et al. 2021).

Genomic and phenotypic analyses confirmed substantial production of IAA, a key plant auxin that regulates root development and overall growth. The synthesis of IAA, a crucial plant auxin, in both plants and microbes is thought to involve multiple pathways, such as tryptamine, IAN, IAM, and IPA as key pathway (Spaepen and Vanderleyden 2011; Kunkel et al. 2024). IAA can act as a signalling molecule, potentially influencing gene expression and biosynthesis in microbes. Consequently, the production of auxin signals by these microbes might be linked to plant defense mechanisms against pathogens (Fadiji et al. 2023). The *ipdC* and *ppdC* genes present at Mz49 genome play a role in transforming tryptophan into IAA via the most prominant IPA pathway. Furthermore, the *amiE* gene, connected to the IAM pathway, may help produce IAA by breaking down IAM into IAA. The Mz49 genome contains *miaABE* genes, which are important for cytokinin production and modification. Similar to *Enterobacter mori* AYS9, which expresses cytokinin genes like *miaA* and *miaB* to convert *Ipr* into 2-methylthio-N6-(dimethylallyl) adenosine and then 2-methylthio-cis-ribozeatin (Fadiji et al. 2023).

The ability of microbes to convert atmospheric nitrogen into a form usable by plants relies on specific enzymes and associated genes (Fadiji et al. 2023). Strain Mz49 was identified to possess the nitrite reductase enzyme, encoded by the *nirB* and *nirD* genes, which play a role in the dissimilatory nitrate reduction pathway. Additionally, genes involved in ammonia (*amtB*) and urea (*ureABCDEFGJ*) transport were detected, potentially contributing to increased nitrogen availability in the soil. The ability of strain Mz49 to dissolve phosphate was confirmed by identifying several genes related to phosphate solubilization and transport. GA recognized as a precursor that promotes phosphate solubilization, is produced by various bacteria. The production of GA is influenced by the enzyme glucose-1-dehydrogenase and the nonprotein chemical pyrroloquinolone quinine (*pqq*). Mz49 genome contained *pqqIFL* genes, studies have shown that the diazotrophic bacterial endophyte *Herbaspirillum seropedicae* Z67, associated with commercial plants, expresses heterologous *pqq* genes (*pqqABCDEF*) that demonstrate phosphate solubilization potential (Wagh et al. 2014). Strain Mz49, also has a higher capacity to absorb phosphate due to the presence of phosphate transport genes (*pstIPN*), which have a high affinity for phosphates. The presence of *pst* genes can improve phosphorus absorption in soil and enhance plant bioavailability. Additionally, the *phoA* gene, which encodes for alkaline phosphatase involved in phosphate assimilation, was also found in strain Mz49, supporting previous findings in *Burkholderia multivorans* WS-FJ9 (Liu et al. 2020). Furthermore, the identification of *ppx* in the Mz49 genome encoding exopolyphosphatase, could influence the strain’s phosphate solubilization ability in the plant rhizosphere. These results are in agreement with Nascimento et al. (2020), who found that the *Bacillus megaterium* STB1 genome contains *phoAD* genes that enhance phosphate solubilization.

One of the central objectives of this study was to identify and characterize genes responsible for phytase activity. Phytate (myo-inositol hexakisphosphate) constitutes the principal storage form of phosphorus in many plant tissues, yet it remains largely unavailable to plants due to the absence of endogenous phytase activity in most crops. Microbial phytases, particularly those produced by PGPR, play a crucial role in the mineralization of phytate, converting it into free phosphate and myo-inositol, thus enhancing phosphorus availability in the rhizosphere. In this context, Mz49 exhibits promising phytase-degrading capabilities that support its function as a biofertilizer.

Our analysis revealed two key genes *agp* (phytase) and *suhB* (inositol monophosphatase), that likely contribute to this function. While the presence of these genes in Enterobacteraceae is not unprecedented, their simultaneous occurrence in a strain exhibiting strong phenotypic phytase activity suggests functional relevance. This dual enzymatic capability may enable efficient dephosphorylation of phytate and subsequent recycling of inositol phosphates, positioning Mz49 as an effective biofertilizer candidate for phosphorus-limited soils. A recent study on novel soil-derived bacteria, *Klebsiella pneumonia* CP-84 and *Chryseobacterium* sp. CP-77, identified the genes responsible for their ability to break down phytate. Specifically, *K. pneumonia* CP-84 was found to contain genes for both a 3-phytase and a glucose-1-phosphatase, the latter also exhibiting phytase activity. In contrast, *Chryseobacterium* sp. CP-77 was shown to possess a gene for a 3-phytase enzyme, which phylogenetic analysis placed within the BPP family. These enzymes represent novel genetic entities, highlighting the potential for discovering unique microbial biocatalysts from previously unexplored environments (Maldonado-Pava et al. 2024).

Recent evidence highlights hidden phytase capacities in related taxa, reinforcing the importance of such traits for sustainable agriculture. In *Enterobacter* species, various phytases have been identified and characterized, each exhibiting distinct properties and potential applications. In a study by Kalsi and co-workers (Kalsi et al. 2016), *E. cloacae* strain PSB-45, a phytase was identified with optimal activity between pH 3.0 and 8.0 and temperatures ranging from 50°C to 70°C, suggesting its suitability for applications in food and feed industries to enhance mineral bioavailability. Similarly, *Enterobacter aerogenes* isolated from hydrocarbon-contaminated soils exhibited extracellular phytase activity. The enzyme reached maximum activity under optimal conditions of pH 5.5 and 50°C after 48 h of incubation. Interestingly, this phytase inhibited biofilm formation in various bacteria and enhanced the degradation of hydrocarbons, indicating its potential in bioremediation applications (Muslim et al. 2018). Comprehensive in silico analysis of the phytase on our study reveals a stable, hydrophilic enzyme with desirable functional features, and its integration into carbohydrate metabolism via protein interaction networks illustrates its biological significance. The physicochemical profile; (acidic nature (pI < 7), moderate molecular weight (~ 48 kDa), low GRAVY values, and favorable aliphatic index) aligns well with characteristics reported for *Enterobacter* sp. phytases in previous bioinformatics analysis (Pramanik et al. 2018). Secondary structure modeling via Phyre2 indicates a predominance of α-helices relative to β-strands and minimal disorder, suggesting a well-folded, stable catalytic fold similar to those seen in other bacterial phytases. This echoes observations from broader phytase studies, where structured α/β architectures are common among active enzymes (Puhl et al. 2007). Embedding the phytase within a STRING-derived interaction network revealed strong associations with key carbohydrate metabolism enzymes; phosphoglucomutase, glucokinase, aldose 1-epimerase, suggesting that phytase-mediated phosphate liberation is closely poised to support glycolysis, glycogen processing, and energy flux. These interaction patterns support a functional model in which phytase activity not only enhances phosphate availability but also interfaces with central metabolic pathways. In line with prior work on microbial phytases, such integration may enhance microbial adaptability and ecological utility, particularly in soil–plant systems where phosphorus mobilization benefits both microbes and hosts (Liu et al. 2022). Taken together, the convergence of stable in silico structural predictions, favorable physicochemical traits, and strategic metabolic interactions strongly supports the potential functional efficacy of this phytase in phosphorus turnover (Rizwanuddin et al. 2023). By reducing reliance on synthetic phosphate fertilizers, strains like Mz49 can improve phosphorus use efficiency and decrease environmental loading. However, to fully establish its novelty and agronomic potential, future work should include comparative genomic analysis of phytase operons, enzymatic kinetics under rhizospheric conditions, and maize growth trials in phosphorus-deficient soils.

Rhizobacteria's ability to suppress plant diseases relies on their production of biocontrol agents. These bacteria employ indirect mechanisms, creating metabolic compounds to combat phytopathogens (Morales-Cedeño et al. 2021). Strain Mz49's biocontrol and stress-reducing capabilities may be acquired from its siderophore production, which enhances its ability to inhibit plant pathogens (Maheshwari et al. 2019). Siderophores are well-known for their role in enhancing plant iron acquisition by solubilizing and transporting iron in the rhizosphere, thereby improving plant growth (Ahmed and Holmström, 2014). Iron is an essential micronutrient required for chlorophyll synthesis and enzymatic functions, and siderophore-producing bacteria have been extensively reported to support plant growth under iron-deficient conditions (Khan et al. 2019). Additionally, the identification of arylpolyene and O-antigen-related gene clusters suggests a role in stress tolerance and protective mechanisms. Arylpolyenes are structurally similar to carotenoids and contribute to oxidative stress resistance, protecting bacterial and plant cells from ROS (Schöner et al. 2016).This function is particularly relevant under drought and other abiotic stress conditions, where oxidative stress can impair plant cellular integrity and metabolism. Genes involved in siderophore biosynthesis and transport, including *fbpABC*, *feoABC*, *fepABCDEG*, and *entABCDEFGHS*, enable the synthesis of enterobactin and aerobactin, which facilitate iron acquisition in the rhizosphere. By solubilizing and transporting iron, these siderophores support chlorophyll synthesis, enzymatic activity, and overall plant growth, particularly under iron-limited conditions. Those genes were identified in strain Mz49, as observed in previous research (Hubrich et al. 2021). The amount of polyamines inside cells is regulated by their synthesis, degradation, removal, and uptake from the surroundings (Kurihara et al. 2011). The conversion of L-arginine to putrescine is facilitated by arginine decarboxylase and agmatinase, enzymes encoded by the *speAB* genes. Additionally, L-ornithine can be transformed into putrescine by ornithine-decarboxylase enzymes, which are coded for by either *speC* or *speF* (Kurihara et al. 2011; Schneider and Wendisch 2011). Spermidine production, a process requiring the *speD* and *speE* enzymes, which were all found in this Mz49 strain.

Beyond hormone and siderophore production, Mz49 contains genes for polyamine biosynthesis, osmoprotectants, and secondary metabolites, including arylpolyenes, NRPs, and thiopeptides, which collectively enhance tolerance to oxidative, osmotic, and temperature stresses. Genome analysis using antiSMASH identified four BGCs encoding siderophores, arylpolyenes, NRPs, and thiopeptides, highlighting Mz49’s capacity to both enhance plant growth and provide protective functions. Arylpolyenes, structurally similar to carotenoids, contribute to oxidative stress resistance, while NRPS- and T1PKS-derived metabolites exhibit antimicrobial activity, collectively supporting pathogen suppression and plant resilience (Majeed et al. 2018).

The integration of genomic and metabolomic data provides a systems-level view of how Mz49 produces VOCs with potential plant growth-promoting properties. Pathway analysis revealed key enzymatic steps for central carbon metabolism and VOC biosynthesis, notably EC 2.2.1.6 (acetolactate synthase, ALS) and EC 1.1.1.4 (butanediol dehydrogenase/acetoin reductase), which form the core of the 2,3-butanediol fermentation route. Genomic evidence confirmed the presence of *ilvH* and *ilvN*,encoding ALS subunits, committing pyruvate to α-acetolactate, a precursor for branched-chain amino acids and acetoin/2,3-butanediol. Furthermore, putative *bdh/adh*-like genes) encode enzymes aligned with EC 1.1.1.4, mediating acetoin to 2,3-butanediol interconversion.

The GC–MS metabolomic profile corroborates these genomic predictions, with 2,3-butanediol detected as a dominant metabolite. Additional VOCs including alcohols, aldehydes, and ketones can be traced to upstream nodes involving thiolase (EC 2.3.1.19), acyl-CoA synthetase (EC 6.2.1.16), and aldehyde dehydrogenase (EC 1.2.1.57), which are also represented in the genome through multiple alcohol/aldehyde dehydrogenase genes. Phenolic/aryl-type VOCs correspond to biosynthetic gene clusters such as arylpolyenes, implicated in oxidative stress protection and signaling (Vincze et al. 2024).

**Fig. 8:**
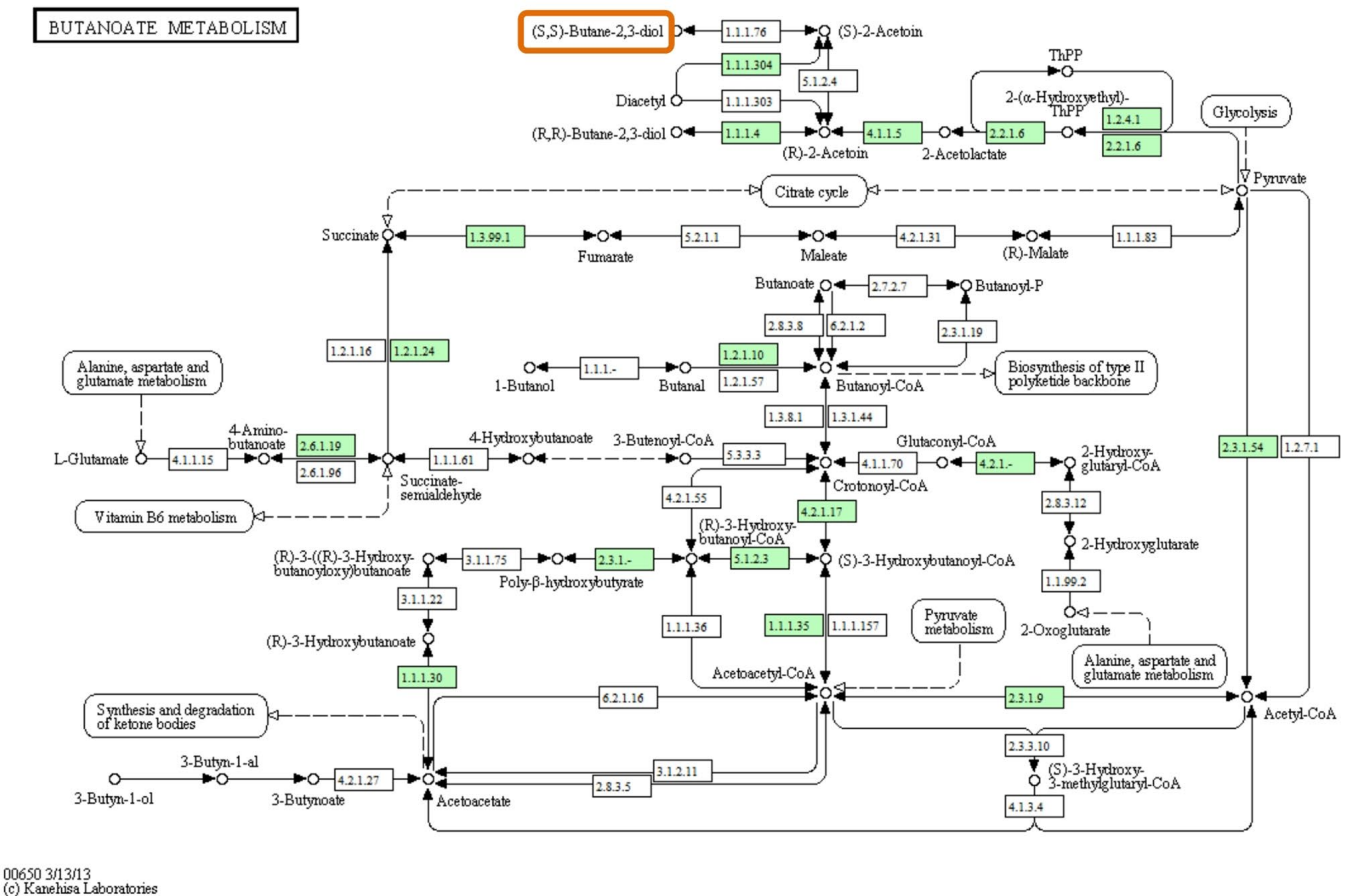
KEGG pathway mapping of VOC biosynthesis in Mz49, highlighting key enzymes and corresponding genes detected in the genome. Core steps include EC 2.2.1.6, Acetolactate synthase (ALS) (*ilvHN)* committing pyruvate to α-acetolactate, and EC 1.1.1.4, Butanediol dehydrogenase/acetoin reductase catalyzing acetoin to 2, 3-butanediol conversion. Pathway visualization integrates genomic annotations (green boxes) and GC–MS-confirmed VOCs (orange box)

This multi-omics alignment highlights a functional metabolic framework where genomic capacity (*als*, *bdh*, *adh* genes) translates into active metabolite production (2,3-butanediol, short-chain alcohols, phenolics) detected via GC–MS, suggesting a direct link between the genetic potential of Mz49 and its plant-beneficial phenotypes, including induction of systemic resistance and enhanced drought tolerance (Cappellari et al. 2020; Li et al. 2021; Kumar et al. 2023). GC–MS profiling confirmed the detection of several VOCs with known roles in plant growth stimulation and pathogen suppression, including alcohols, ketones, and aromatic compounds. While the current discussion of VOC biosynthetic pathways is limited, metabolomic signatures are integrated with corresponding genomic loci (e.g., genes for acetoin, 2, 3-butanediol synthesis). Such VOCs can promote systemic resistance in plants and modulate root architecture, suggesting an additional layer through which Mz49 enhances maize performance under stress (Fig. 8).

Furthermore; metabolic profiling using GC–MS confirmed that Mz49 produces another bioactive compounds with growth-promoting and stress-alleviating roles. For instance, 2, 3-butanediol stimulates root development and stress tolerance, succinic and palmitic acids (Zhukov, 2015) enhance root growth and energy metabolism (Li et al. 2021)., D-pinitol maintains cellular integrity under drought and salinity (Ahn et al. 2018)., and tyrosol provides antioxidant protection (Silva et al. 2021).. Furthermore, azelaic acid induces systemic acquired resistance (Haghpanah et al. 2024, Priy 2024), while hexanoic, octanoic, and propanoic acids exert antimicrobial effects (Gāliņa et al. 2022) collectively reducing pathogen pressure (Dijksterhuis et al. 2019). Together, siderophore production, secondary metabolites, and polyamine synthesis enable Mz49 to simultaneously promote plant growth and enhance resistance to biotic and abiotic stresses, making it a versatile and effective rhizobacterium for sustainable agriculture.

Rhizobacteria strain Mz49 exhibits gene activity related to the production of both IAA and siderophores, aligning with existing research on PGP characteristics in rhizobacteria. IAA production was further confirmed by phenotypic assay where Mz49 produced considerable amounts of IAA, cytokinin production appears to be essential for promoting plant health and growth (Wani et al. 2016). Furthermore, the presence of siderophore and iron-related genes in Mz49 suggests a potential for improved plant development by enhancing access to soil minerals. Iron transport genes in Mz49 may also play a key role in making insoluble iron available for plant uptake.

In all living organisms, glutamine and glutamate play crucial roles as primary donors of amino groups for nitrogen-containing compounds, including other amino acids and the components necessary for RNA and DNA synthesis. The strain Mz49 possesses genes such as *gltBDSIJKLS*, which are involved in glutamate production and transport. Glutamate not only participates in anabolic processes but also functions as an intracellular antagonist to potassium. Furthermore, it serves as an osmoprotectant in certain bacteria and archaea (Saum et al. 2006). Our results align with previous research on *B. subtilis*, where glutamate is identified as a precursor to proline, which accumulates in high concentrations under hyperosmotic conditions and acts as a protective solute for cells.

The heavy metal resistance mechanisms exhibited by Mz49 may also have a significant role in plant growth promotion, particularly in metal-contaminated soils or environments where high concentrations of heavy metals inhibit plant growth. Several studies have demonstrated the potential of metal-resistant bacteria to assist in plant growth by inhibiting metal toxicity and enhancing nutrient availability (Glick, 2012). The strain Mz49 also exhibits a complex array of genetic mechanisms conferring resistance to various heavy metals, including antimony, arsenic, cobalt, copper, iron, manganese, mercury, nickel, selenium, and zinc. These resistance mechanisms are primarily mediated by metal transport systems and efflux pumps. For antimony and arsenic, genes such as *arsB* and *arsC1*, along with *pitA* and *pstABCS*, regulate the import and export of these toxic ions (Ben Fekih et al. 2018). Copper resistance involves efflux systems encoded by *cusABC*, and regulatory genes like *cusRS* that help remove excess copper ions (Nies, 2003). Manganese homeostasis is controlled by *mntR*, *dtxR*, and *sirR*, while transporters such as *mntH* mediate manganese uptake and efflux. Mercury resistance is regulated by the *exoR* gene, while nickel resistance involves genes such as *ddpA* and *rcnA* for uptake and homeostasis (Silver and Phung 2005). Selenium resistance is facilitated by genes like *selA* and *ynfE* (Sierra et al., 2015), and silver resistance is mediated by the Cus efflux system, including *cusA* and *cusF* (Franke et al. 2003). Zinc resistance is regulated by transporters like *zntA* and *znuA* (Hussain et al. 2022). Additionally, the *cutA* gene contributes to general tolerance to divalent cations, and the TonB-ExbB-ExbD system facilitates nutrient uptake (Noinaj et al. 2010). This study represents a preliminary genomic and metabolic characterization of Mz49. While in silico analysis provided insights into potential phytase activity, stress tolerance, and other PGP traits, direct functional validation remains limited. The detection of virulence factors and antibiotic resistance genes within the Mz49 genome necessitates careful risk assessment. Although these elements are common among environmental *Enterobacter* strains, their presence raises concerns about horizontal gene transfer and opportunistic pathogenicity. The high PathogenFinder score reinforces the need for functional assays to confirm pathogenic potential. Future studies will focus on quantifying phytase activity, conducting greenhouse or field trials, and experimentally correlating genomic predictions, such as heavy metal resistance operons, with phenotypic assays. These steps will be essential to confirm the functional potential and biosafety of Mz49 for agricultural applications.

## Conclusion

In conclusion, the genomic and metabolic analysis of *E. cloacae* strain Mz49, isolated from the maize rhizosphere, highlights its potential in plant growth promotion and stress tolerance. The strain's genome contains a diverse array of genes involved in producing growth hormones, phytate degradation, VOCs, siderophores, cold and heat shock proteins, trehalose, glycine betaine, phenazine, and NRPs. These genetic features enable Mz49 to enhance plant resistance to environmental stressors and promote plant health under adverse conditions. The biotechnological significance of these genes is crucial, offering promising solutions for improving plant resilience to heavy metals, biotic and abiotic stresses, enhancing soil health, and crop yields. This genomic analysis reveals the potential of Mz49 in sustainable agriculture. By prioritizing phytase activity, stress adaptation, and PGPR traits, Mz49 emerges as a promising candidate for sustainable maize cultivation in stress-prone environments. However, practical application relies on addressing biosafety concerns, integrating omics data into predictive models, and validating these findings under real-world agronomic conditions. Future research should focus on studying these genetic capabilities to develop effective bioinoculants for addressing abiotic stress-related challenges in agriculture.

## Supplementary Information

Supplementary file 1 (XLSX 6 kb)

Supplementary file 2 (DOCX 19 kb)

## Data Availability

The original contributions presented in the study are included in the article, further inquiries can be directed to the corresponding author.
