# Supplementary material for: Genomic prediction of phytase potential and stress tolerance in maize-associated plant growth-promoting rhizobacterium Enterobacter cloacae Mz49
Source: AMB Express. 2025 Nov 29;15:180. doi: 10.1186/s13568-025-01981-8 (PMC12712290; doi:10.1186/s13568-025-01981-8)
Supplement: Supplementary file 2 — (DOCX 19 kb) [file 13568_2025_1981_MOESM2_ESM.docx]

Supplementary Figures


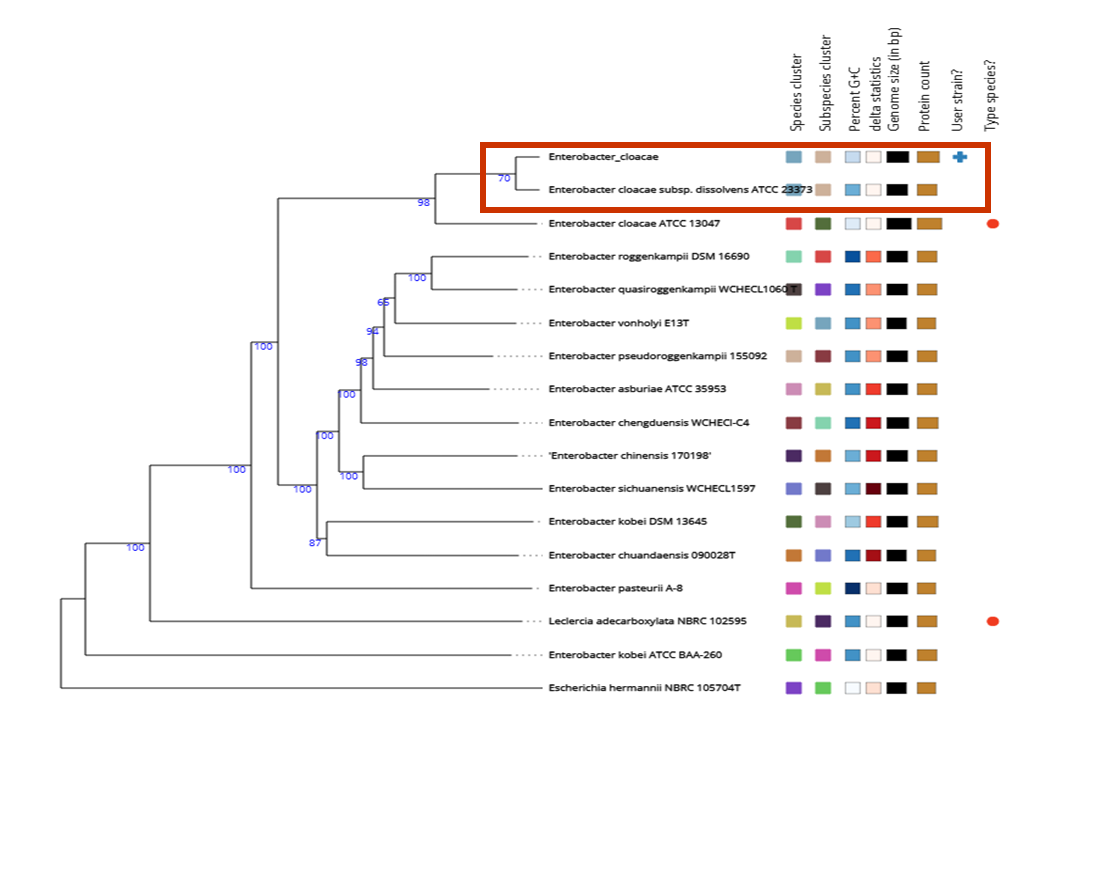
**Fig. S1.** **Phylogenomic tree constructed from GBDP distances calculated from the genomes of the TYGS type strains most closely related to Mz49.** Species and subspecies clusters are defined by a 70% and 79% dDDH thresholds, respectively. The numbers above the branches represent GBDP pseudo-bootstrap support values from 100 replicates. The tree is midpoint-rooted.


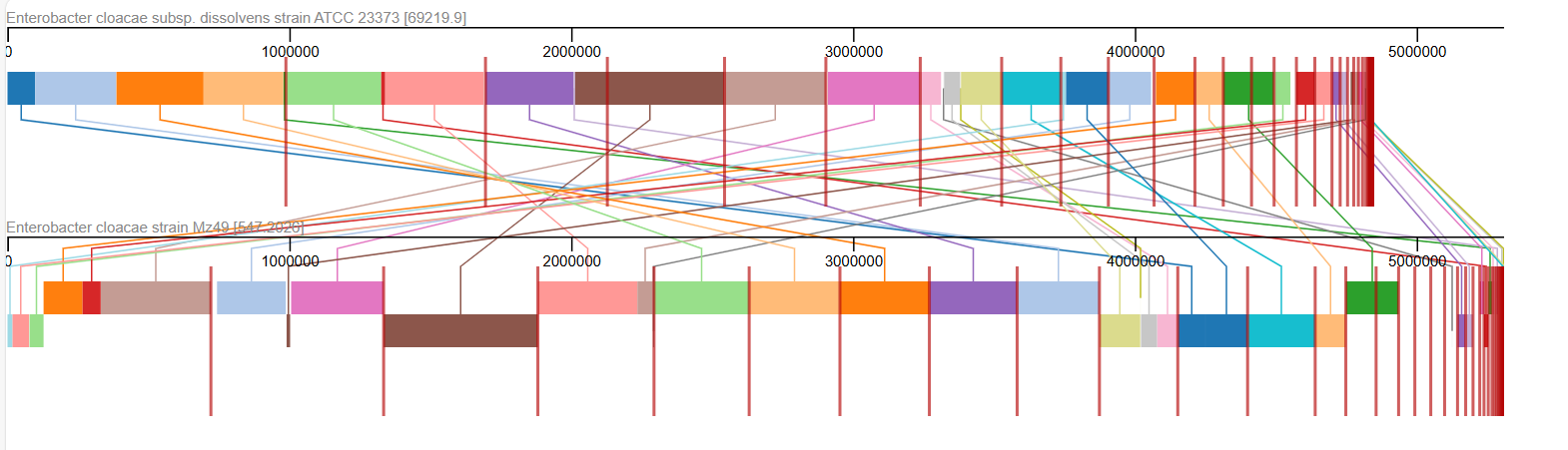


**Fig. S2. *E. cloacae* subsp. *dissolvens* ATCC 23373T and Mz49 genomes are compared using Mauve 2.3.1 software**


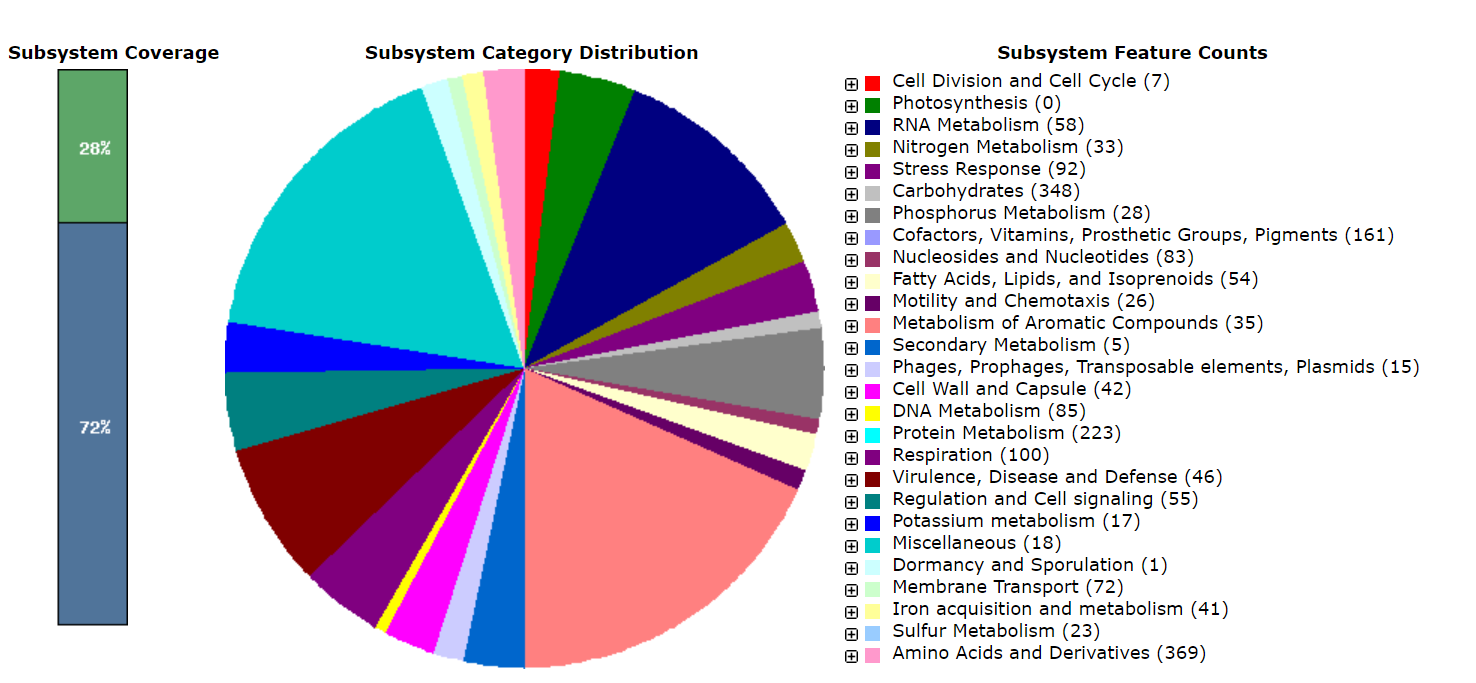


**Fig. S3. SEED subsystems classification of the genome of Mz49 predicted by the RAST annotation server.** The subsystem coverage bar classifies predicted proteins into those within subsystems (green) and those outside subsystems (blue). The subsystem category distribution pie chart shows the percentage of predicted proteins across different pathways, each represented by a unique color. The number of predicted subsystems for each feature is shown in parentheses.


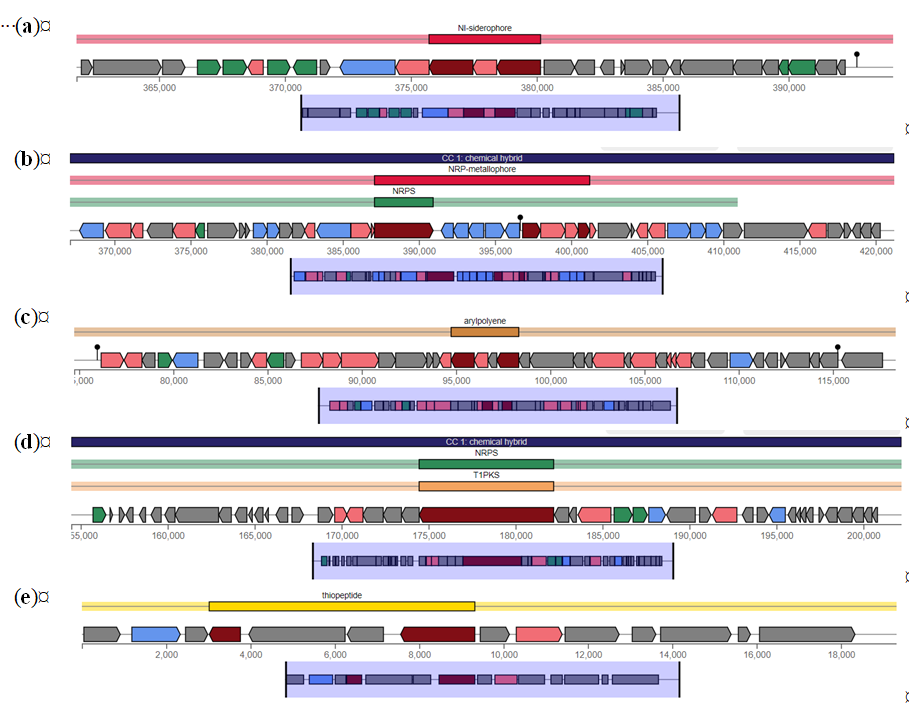


**Fig. S4. BGCs identified in the genome of Mz49 using antiSMASH version 7.1.0.,** (a) NI-siderophore (aerobactin), (b) NRP-metallophore, NRPS (enterobactin), (c) arylpolyene, (d) NRPS,T1PKS, and (e) thiopeptide (O-antigen)

**
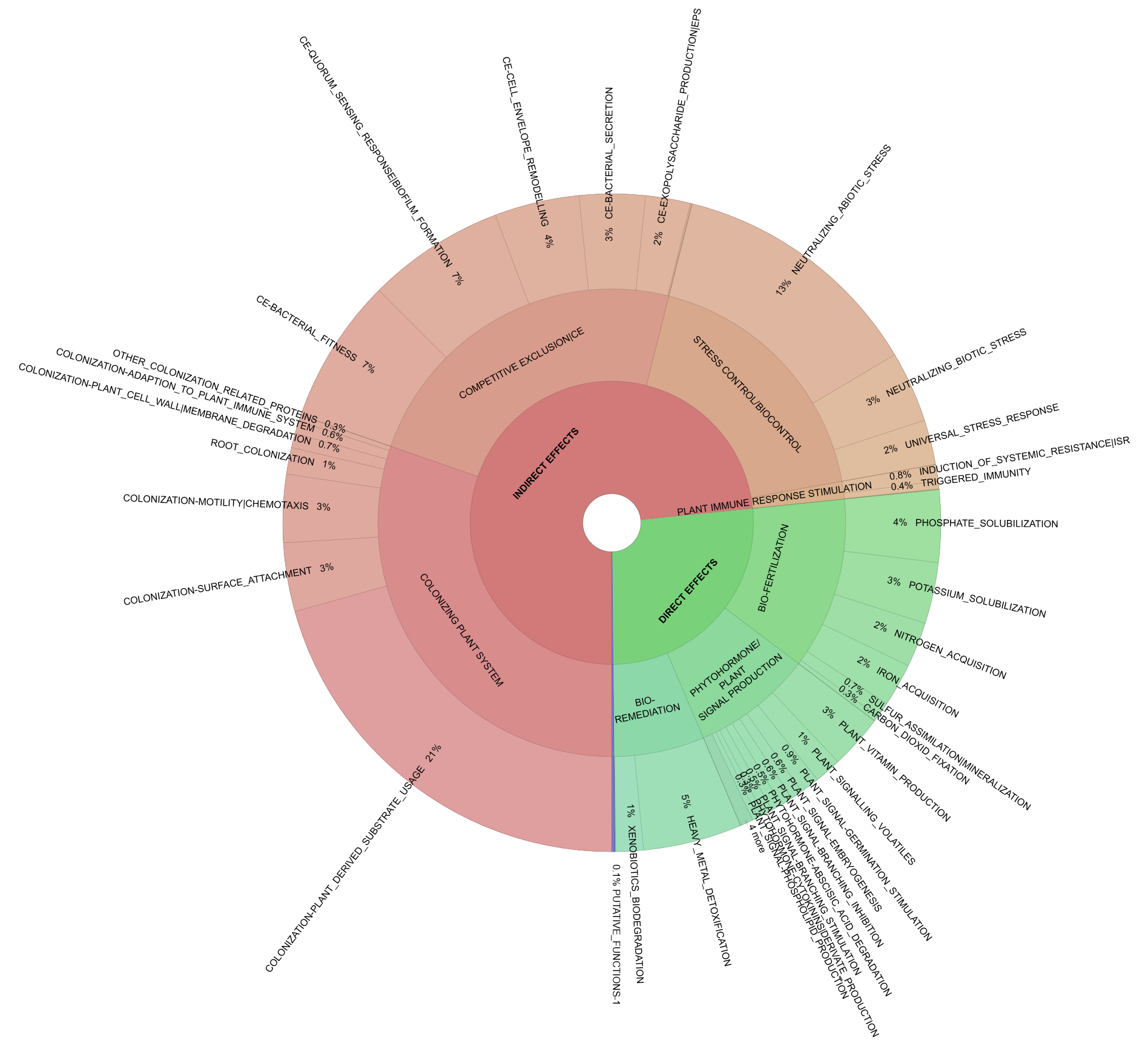
Fig. S5. Plant-related genes determined by the PGPT-Pred tool from PLaBAse**


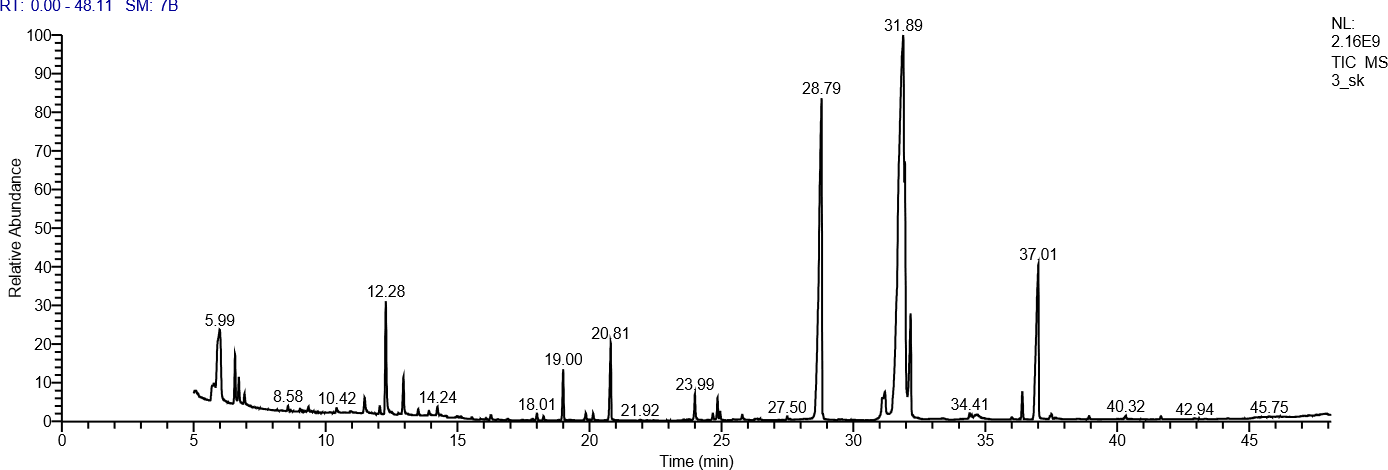

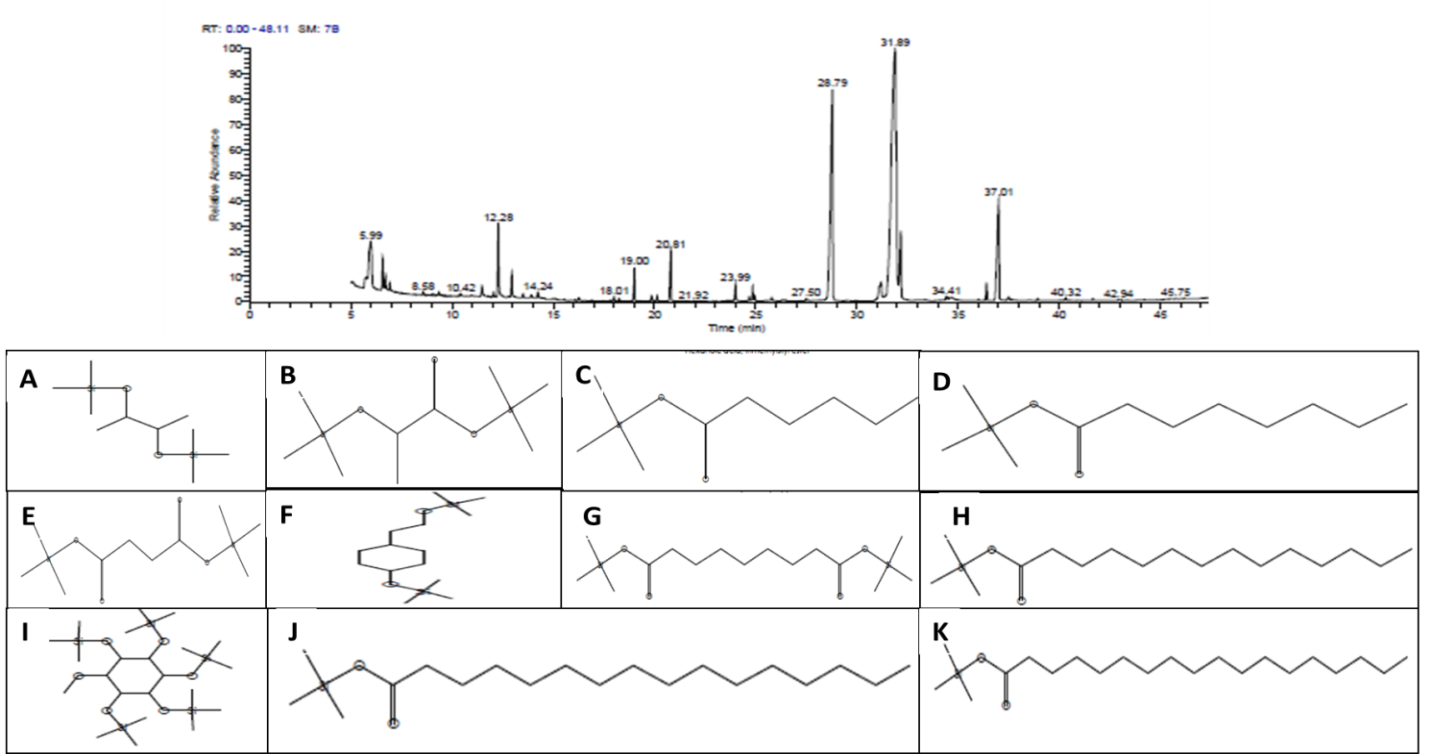


**Fig. S6. GC–MS analysis of the CFS of Mz49.** Identified compounds: (A) 2,3-Butanediol; (B) Propanoic acid (C) Hexanoic acid; (D) Octanoic acid; (E) Butanedioic acid; (F) Tyrosol; (G) Azelaic acid; (H) Myristic acid; (I) D-pinitol, (J) Palmitic acid; and (K) Stearic acid.

cov pid  **1** **[ . . . . : . . .** **80**

1 Enterobacter_cloacae_Mz49[JBGMDT000000000] 100.0% 100.0% **MRKALLAVAVAGLFSAASGVQAQETPEGYQLEQVLIMSRHNLRAPLANNGSVLEQSTPKQWPEWEVPGGQLTTKGGVLEV**

2 Enterobacter_sp.[WP_014831381.1] 100.0% 100.0% **MRKALLAVAVAGLFSAASGVQAQETPEGYQLEQVLIMSRHNLRAPLANNGSVLEQSTPKQWPEWEVPGGQLTTKGGVLEV**

3 Enterobacter_cloacae[WP_248086903.1] 100.0% 99.8% **MRKALLAVAVAGLFSAASGVQAQETPEGYQLEQVLIMSRHNLRAPLANNGSVLEQSTPKQWPEWEVPGGQLTTKGGVLEV**

4 Enterobacter_cloacae[WP_335277789.1] 100.0% 99.8% **MRKALLAVAVAGLFSAASGVQAQETPEGYQLEQVLIMSRHNLRAPLANNGSVLEQSTPKQWPEWEVPGGQLTTKGGVLEV**

5 Enterobacter_cloacae[WP_368529435.1] 100.0% 99.8% **MRKALLAVAVAGLFSAASGVQAQETPEGYQLEQVLIMSRHNLRAPLANNGSVLEQSTPKQWPEWEVPGGQLTTKGGVLEV**

6 Enterobacter_cloacae[WP_262819480.1] 100.0% 99.8% **MRKALLAMAVAGLFSAASGVQAQETPEGYQLEQVLIMSRHNLRAPLANNGSVLEQSTPKQWPEWEVPGGQLTTKGGVLEV**

7 Enterobacter_cloacae[WP_230135050.1] 100.0% 99.3% **MRKALLAVAVAGLFSAASGVQAQETPEGYQLEQVLIMSRHNLRAPLANNGSVLEQSTPKQWPEWEVPGGQLTTKGGVLEV**

8 Enterobacter_sp.[WP_104948910.1] 100.0% 96.9% **MRKALLALAVAGSISATFGVQAQETPEGYQLEQVLIMSRHNLRAPLANNGSVLEQSTPKQWPEWEVPGGQLTTKGGVLEV**

9 Enterobacter_vonholyi[WP_133294140.1] 100.0% 96.1% **MRKALLALAVAGSISATFGVQAQETPEGYQLEQVLIMSRHNLRAPLANNGSVLEQSTPKQWPEWEVPGGQLTTKGGVLEL**

10 Enterobacter_roggenkampii[WP_069598106.1] 100.0% 96.1% **MRKALLALAVAGSISATFGVQAQETPEGYQLEQVLIMSRHNLRAPLANNGSVLEQSTPKQWPEWEVPGGQLTTKGGVLEV**

11 Enterobacter_quasimori[WP_216357052.1] 100.0% 95.9% **MRKALLAVAVAGTLSVTFGAQAQETPDGYQLEQVLIMSRHNLRAPLANNGSVLEQSTPKQWPEWEVPGGQLTTKGGVLEV**

12 Enterobacter_sp.[WP_126328197.1] 100.0% 96.1% **MRKALLALAVAGSISATFGVQAQETPEGYQLEQVLIMSRHNLRAPLANNGSVLEQSTPKQWPEWEVPGGQLTTKGGVLEV**

13 Enterobacter_sp.[WP_200557788.1] 100.0% 95.4% **MRKALLALAVAGSISATFGVQAQETPEGYQLEQVLIMSRHNLRAPLANNGSVLEQSTPKQWPEWEVPGGQLTTKGGVLEV**

14 Enterobacter_sp.[WP_049008444.1] 100.0% 94.9% **MRKALLALAIAGSISATFGVQAQETPEGYQLEQVLIMSRHNLRAPLANNGSVLEQSTPKQWPEWEVPGGQLTTKGGVLEV**

15 Enterobacter_bugandensis[WP_088204980.1] 100.0% 95.2% **MRKALLAVAVAGTLSMTFGAQAQETPKGYQLEQVLIMSRHNLRAPLANNGSVLEQSTPKQWPEWDVPGGQLTTKGGVLEV**

16 Enterobacter_mori[WP_237847005.1] 100.0% 95.4% **MRKALLAVAVAGTLSVTFGAQAQETPDGYQLEQVLIMSRHNLRAPLANNGSVLEQSTPKQWPEWEVPGGQLTTKGGVLEV**

17 Enterobacter_pseudoroggenkampii[WP_290094731.1] 100.0% 95.4% **MRKALLALAVAGSISATFGVQAQETPEGYQLEQVLIMSRHNLRAPLANNGSVLEQSTPKQWPEWEVPGGQLTTKGGVLEV**

18 Enterobacter_chuandaensis[WP_119914887.1] 100.0% 95.2% **MRKALLALAVAGSISATFGVQAQETPEGYQLEQVLIMSRHNLRAPLANNGSVLEQSTPKQWPEWEVPGGQLTTKGGVLEV**

19 Enterobacter_sichuanensis[WP_059385533.1] 100.0% 94.7% **MRKALLALAVAGSISATFGVQAQETPEGYQLEQVLIMSRHNLRAPLANNGSVLEQSTPKQWPEWEVPGGQLTTKGGVLEV**

20 Enterobacter_nematophilus[WP_266178332.1] 100.0% 94.4% **MRKALLAVAVAGTLSMTFGAQAQETPEGYQLQQVLIMSRHNLRAPLANNGSVLEQSTPKQWPEWDVPGGQLTTKGGVLEV**

21 Enterobacter_ludwigii[WP_040017112.1] 100.0% 93.9% **MRKALLALAIAGSITVTSGVQAQETPEGYQLEQVLIMSRHNLRAPLANNGSVLEQSTPKQWPEWEVPGGQLTTKGGVLEV**

22 Enterobacter_wuhouensis[WP_326471074.1] 100.0% 93.5% **MRKALLAVAVAGSLSLTFAVQAQDTPDGYQLEQVLIMSRHNLRAPLANNGSVLEQSTPKQWPEWEVPGGQLTTKGGVLEV**

23 Enterobacter_huaxiensis[WP_119934760.1] 100.0% 93.2% **MRKALLAVAVAGSLSLTFGAQAQDTPDGYQLEQVLIMSRHNLRAPLANNGSVLEQSTPKQWPEWEVPGGQLTTKGGVLEV**

24 Silvania_confinis[WP_271269508.1] 100.0% 85.5% **MRKALIAAAVAGSIGLSCGVQAQTAPEGYQLQQVLIMSRHNLRAPLANNGSVLEQSTPSKWPEWDVPGGQLTTKGGVLEV**

25 WP_262664223.1 100.0% 85.5% **MRKALIAVAVAGSIGLSGGVQAQTAPEGYQLEQVLIMSRHNLRAPLANNGSVLEQSTPEKWPEWDVPGGQLTTKGGVLEV**

26 Citrobacter_sp.[WP_045441506.1] 100.0% 84.3% **MKKSLIAAAVAGVVLLSSAAQAQTTPEGYQLQQVLMMSRHNLRAPLANNGSVLEQSTPKQWPQWEVPGGQLTTKGGVLEI**

27 Silvania_hatchlandensis[WP_271281829.1] 100.0% 84.5% **MRKALIAAAVAGSIGLSCGVQAQTAPDGYQLEQVLIMSRHNLRAPLANNGSVLEQSTPSKWPEWDVPGGQLTTKGGVLEV**

28 Citrobacter_rodentium[WP_012905376.1] 100.0% 83.3% **MKKTLIAAAVAGVVMLSSGAQAQTTPEGYQLQQVLMMSRHNLRAPLANNGSVLEQSTPNKWPEWDVPGGQLTTKGGVLEV**

29 Citrobacter_farmeri[WP_042319761.1] 100.0% 83.3% **MNKSLIAAAVAGVVMLSSAAQAQTAPEGYQLQQVLMMSRHNLRAPLANNGSVLEQSTPNKWPEWDVPGGQLTTKGGVLEV**

30 Pseudenterobacter_timonensis[WP_061708644.1] 100.0% 83.1% **MRKAPLVSAVAGMLALVSGAQAQNTPEGYQLQQVLIMSRHNLRAPLADNGSVLAQSTPNEWPQWDVPGGQLTTKGGVLEV**

31 Citrobacter_sp.[WP_038639533.1] 100.0% 83.8% **MNKSLIAAAVAGAVLVSGAAYAQTAPEGYQLQQVLMMSRHNLRAPLANNGSVLEQSTPKQWPEWDVPGGQLTTKGGVLEV**

32 Citrobacter_freundii[WP_201506408.1] 100.0% 83.1% **MNKSLIAAAVAGAVLLSNAAQAQTAPEGYQLQQVLMMSRHNLRAPLANNGSVLEQSTPKQWPEWDVPGGQLTTKGGVLEI**

33 Pseudescherichia_vulneris[WP_154057810.1] 95.2% 86.8% **--------------------QAQTTPEGYQLQQVLIMSRHNLRAPLADNGSVLQQSTPNKWPEWDVPGGQLTTKGGVLEV**

34 Citrobacter_telavivensis[WP_373671151.1] 100.0% 83.1% **MNKSLIAAAVAGVVMLSSAAQAQAAPEGYQLQQVLMMSRHNLRAPLANNGSVLEQSTPNKWPEWDVPGGQLTTKGGVLEV**

35 Scandinavium_goeteborgense[WP_125354742.1] 100.0% 81.8% **MRKSVLAVAIIGSMMAGFAAQAETTPEGYQLQQVLIMSRHNLRAPLANNGSVLEQSTPNKWPEWDVPGGQLTTKGGVLEV**

consensus/100% **.....................AptsPcGYQLpQVLhMSRHNLRAPLAsNGSVLtQSTPppWPpW-VPGGQLTTKGGVLEl**

consensus/90% **MpKuLlAhAlAG.h.hs.usQAQpsP-GYQLpQVLhMSRHNLRAPLANNGSVLEQSTPppWPEW-VPGGQLTTKGGVLEV**

consensus/80% **MRKuLlAhAVAG.h.hs.usQAQpTPEGYQLpQVLIMSRHNLRAPLANNGSVLEQSTPppWPEW-VPGGQLTTKGGVLEV**

consensus/70% **MRKALLAhAVAGsluho.GsQAQpTPEGYQLpQVLIMSRHNLRAPLANNGSVLEQSTPKQWPEW-VPGGQLTTKGGVLEV**

cov pid  **81**  **. 1 . . . . : .** **160**

1 Enterobacter_cloacae_Mz49[JBGMDT000000000] 100.0% 100.0% **YMGHYMREWLAQQGMVKTGECPPAESVYAYANSLQRTVATAQFFITGAFPGCDVPVHHQDKMGTMDPTFNPVITDNSPEF**

2 Enterobacter_sp.[WP_014831381.1] 100.0% 100.0% **YMGHYMREWLAQQGMVKTGECPPAESVYAYANSLQRTVATAQFFITGAFPGCDVPVHHQDKMGTMDPTFNPVITDNSPEF**

3 Enterobacter_cloacae[WP_248086903.1] 100.0% 99.8% **YMGHYMREWLAQQGMVKTGECPPAESVYAYANSLQRTVATAQFFITGAFPGCDVPVHHQDKMGTMDPTFNPVITDNSPEF**

4 Enterobacter_cloacae[WP_335277789.1] 100.0% 99.8% **YMGHYMREWLAQQGMVKTGECPPAESVYAYANSLQRTVATAQFFITGAFPGCDVPVHHQDKMGTMDPTFNPVITDNSPEF**

5 Enterobacter_cloacae[WP_368529435.1] 100.0% 99.8% **YMGHYMREWLAQQGMVKTGECPPAESVYAYANSLQRTVATAQFFITGAFPGCDVPVHHQDKMGTMDPTFNPVITDNSPEF**

6 Enterobacter_cloacae[WP_262819480.1] 100.0% 99.8% **YMGHYMREWLAQQGMVKTGECPPAESVYAYANSLQRTVATAQFFITGAFPGCDVPVHHQDKMGTMDPTFNPVITDNSPEF**

7 Enterobacter_cloacae[WP_230135050.1] 100.0% 99.3% **YMGHYMREWLAQQGMVKTGECPPAESVYAYANSLQRTVATAQFFITGAFPGCDVPVHHQDKMGTMDPTFNPVITDNSPEF**

8 Enterobacter_sp.[WP_104948910.1] 100.0% 96.9% **YMGHYMREWLAQQGMVKTGECPPAESVYAYANSLQRTVATAQFFITGAFPGCDVPVHHQEKMGTMDPTFNPVITDNSPEF**

9 Enterobacter_vonholyi[WP_133294140.1] 100.0% 96.1% **YMGHYMREWLAQQGMVKTGECPAADSVYAYANSLQRTVATAQFFITGAFPGCDVPVHHQEKMGTMDPTFNPVITDNSPEF**

10 Enterobacter_roggenkampii[WP_069598106.1] 100.0% 96.1% **YMGHYMREWLAQQGMVKTGECPPADSVYAYANSLQRTVATAQFFITGAFPGCDLPVHHQEKMGTMDPTFNPVITDNSPEF**

11 Enterobacter_quasimori[WP_216357052.1] 100.0% 95.9% **YMGHYMREWLAQQGMVTTGECPPADSVYAYANSLQRTVATAQFFITGAFPGCDVPVHHQEKMGTMDPTFNPVITDNSPEF**

12 Enterobacter_sp.[WP_126328197.1] 100.0% 96.1% **YMGHYMREWLAQQGMVKTGECPAADSVYAYANSLQRTVATAQFFITGAFPGCDVPVHHQDKMGTMDPTFNPVITDNSPEF**

13 Enterobacter_sp.[WP_200557788.1] 100.0% 95.4% **YMGHYMREWLAQQGMIKTGECPPADTVYAYANSLQRTVATAQFFITGAFPGCDVPVHHQEKMGTMDPTFNPVITDNSPEF**

14 Enterobacter_sp.[WP_049008444.1] 100.0% 94.9% **YMGHYMREWLAEQGMVKTGECPPADSVYAYANSLQRTVATAQFFITGAFPGCDIPVHHQEKMGTMDPTFNPVITDNSPEF**

15 Enterobacter_bugandensis[WP_088204980.1] 100.0% 95.2% **YMGHYMREWLAEQGMVKTGECPPADSVYAYANSLQRTVATAQFFITGAFPGCDVPVHHQEKMGTMDPTFNPVITDNSPEF**

16 Enterobacter_mori[WP_237847005.1] 100.0% 95.4% **YIGHYMREWLAQQGMVTTGECPPADSVYAYANSLQRTVATAQFFITGAFPGCDVPVHHQEKMGTMDPTFNPVITDNSPEF**

17 Enterobacter_pseudoroggenkampii[WP_290094731.1] 100.0% 95.4% **YMGHYMREWLAQQGMVKTGECPAADSVYAYANSLQRTVATAQFFITGAFPGCDVPVHHQEKMGTMDPTFNPVITNNSPEF**

18 Enterobacter_chuandaensis[WP_119914887.1] 100.0% 95.2% **YMGHYMREWLAKQGMVKTGECPAADSVYAYANSLQRTVATAQFFITGAFPGCDVPVHHQEKMGTMDPTFNPVITDNSPEF**

19 Enterobacter_sichuanensis[WP_059385533.1] 100.0% 94.7% **YMGHYMREWLAQQGMIKSGECPPADAVYAYANSLQRTVATAQFFITGAFPGCDVPVHHQEKMGTMDPTFNPVITDNSPEF**

20 Enterobacter_nematophilus[WP_266178332.1] 100.0% 94.4% **YMGHYMREWLAEQGMVKTGECPAADSVYAYANSLQRTVATAQFFITGAFPGCDVPVHHQEKMGTMDPTFNPVITDNSPEF**

21 Enterobacter_ludwigii[WP_040017112.1] 100.0% 93.9% **YMGHYMREWLAQQGMVKTGECPAADSVYAYANSLQRTVATAQFFITGAFPGCDVPVHHQEKIGTMDPTFNPVITDNSPEF**

22 Enterobacter_wuhouensis[WP_326471074.1] 100.0% 93.5% **YMGHYMREWLAQQGMVTSGECPPADSVYAYANSLQRTVATAQFFITGAFPGCDIPVHHQEKMGTMDPTFNPVITDNSPEF**

23 Enterobacter_huaxiensis[WP_119934760.1] 100.0% 93.2% **YMGHYMREWLAQQGMVTTGECPPADSVYAYANSLQRTVATAQFFITGAFPGCDIPVHHQEKMGTMDPTFNPVITDNSPEF**

24 Silvania_confinis[WP_271269508.1] 100.0% 85.5% **YMGHYLREWLAEQGMVTAGECPTAGSVYAYANSLQRTVATAQFFITGAFPGCDVPVHHQEQMGTMDPTFNPVITDNSPAF**

25 WP_262664223.1 100.0% 85.5% **YMGHYMREWLAEEGVVTAGECPTANSVYAYANSLQRTVATAQFFITGAFPGCDVPVHHQEQMGTMDPTFNPVITDNSPEF**

26 Citrobacter_sp.[WP_045441506.1] 100.0% 84.3% **YMGHYMREWLAEQGMVKSGECPAPDTVYTYANSLQRTVATAQFFVTGAFPGCDVPVHHQEKMGTMDPTFNPVITDDSAAF**

27 Silvania_hatchlandensis[WP_271281829.1] 100.0% 84.5% **YMGHYLREWLAEQGMVTAGECPTAGSVYAYANSLQRTVATAQFFITGAFPGCDVPVHHQEQMGTMDPTFNPVITDNSPEF**

28 Citrobacter_rodentium[WP_012905376.1] 100.0% 83.3% **YMGHYMREWLAAQGMVKSGECPAPDAVYAYANSLQRTVATAQFFITGAFPGCDVPVHHQEKMGTMDPTFNPVITDDSPTF**

29 Citrobacter_farmeri[WP_042319761.1] 100.0% 83.3% **YMGHYMREWLAEQGMVKSGECPEPETVYTYANSLQRTVATAQFFITGAFPGCDVPVHHQEKMGTMDPTFNPVITDDSAAF**

30 Pseudenterobacter_timonensis[WP_061708644.1] 100.0% 83.1% **YMGHYMREWLAQEGLVRAGECPAPQAVYAYANSLQRTVATAQFFITGAFPGCDVPVHHQEKMGTMDPTFNPVITDNSPAF**

31 Citrobacter_sp.[WP_038639533.1] 100.0% 83.8% **YMGHYMREWLAEQGMVKTGECPAPDTVYTYANSLQRTVATAQFFVTGAFPGCDVPVHHQEKMGTMDPTFNPVITDDSAAF**

32 Citrobacter_freundii[WP_201506408.1] 100.0% 83.1% **YMGHYMREWLAEQGMVKSGECPAPDTVYTYANSLQRTVATAQFFVTGAFPGCDVPVHHQEKMGTMDPTFNPVITDDSATF**

33 Pseudescherichia_vulneris[WP_154057810.1] 95.2% 86.8% **YMGHYMREWLAEQGLVKAGECPTSDAVYAYANSLQRTVATAQFFITGAFPGCDVPVHHQDAMGTMDPTFNPVITDKDAAF**

34 Citrobacter_telavivensis[WP_373671151.1] 100.0% 83.1% **YMGHYMREWLAEQGMVKSGECPAPETVYTYANSLQRTVATAQFFITGAFPGCDVPVHHQEKMGTMDPTFNPVITDDSVAF**

35 Scandinavium_goeteborgense[WP_125354742.1] 100.0% 81.8% **YMGHYMREWLAEKGMVTTGECPPADAVYAYANSLQRTVATAQFFITGAFPGCDVPVHHQDKMGTMDPTFNPVITDSSAAF**

consensus/100% **YhGHYhREWLAtpGhlpsGECP.stsVYsYANSLQRTVATAQFFlTGAFPGCDlPVHHQ-thGTMDPTFNPVITspsstF**

consensus/90% **YMGHYMREWLApQGMVpsGECPsspsVYsYANSLQRTVATAQFFITGAFPGCDlPVHHQ-pMGTMDPTFNPVITDsSstF**

consensus/80% **YMGHYMREWLApQGMVpoGECPsu-oVYAYANSLQRTVATAQFFITGAFPGCDVPVHHQ-KMGTMDPTFNPVITDsSPtF**

consensus/70% **YMGHYMREWLApQGMVKoGECPsA-oVYAYANSLQRTVATAQFFITGAFPGCDVPVHHQEKMGTMDPTFNPVITDNSPEF**

cov pid **161**  **. . . 2 . . . .** **240**

1 Enterobacter_cloacae_Mz49[JBGMDT000000000] 100.0% 100.0% **REKALKAMESERQKMQLGESYKLLEQMTNYADSPSCKEKKVCSLADAKDTFSADYEKEPGVSGPLKVGNSLVDAFTLQYY**

2 Enterobacter_sp.[WP_014831381.1] 100.0% 100.0% **REKALKAMESERQKMQLGESYKLLEQMTNYADSPSCKEKKVCSLADAKDTFSADYEKEPGVSGPLKVGNSLVDAFTLQYY**

3 Enterobacter_cloacae[WP_248086903.1] 100.0% 99.8% **REKALKAMESERQKMQLGESYKLLEQMTNYADSPSCKEKKVCSLADAKDTFSADYEKEPGVSGPLKVGNSLVDAFTLQYY**

4 Enterobacter_cloacae[WP_335277789.1] 100.0% 99.8% **REKALKAMESERQKMQLGESYKLLEQMTNYADSPSCKEKKVCSLADAKDTFSADYEKEPGVSGPLKVGNSLVDAFTLQYY**

5 Enterobacter_cloacae[WP_368529435.1] 100.0% 99.8% **REKALKAMESERQKMQLGESYKLLEQMTNYADSPSCKEKKVCSLADAKDTFSADYEKEPGVSGPLKVGNSLMDAFTLQYY**

6 Enterobacter_cloacae[WP_262819480.1] 100.0% 99.8% **REKALKAMESERQKMQLGESYKLLEQMTNYADSPSCKEKKVCSLADAKDTFSADYEKEPGVSGPLKVGNSLVDAFTLQYY**

7 Enterobacter_cloacae[WP_230135050.1] 100.0% 99.3% **REKALKAMESERQKMQLAESYRLLEQMTNYADSPSCKEKKVCSLADAKDTFSADYEKEPGVSGPLKVGNSLVDAFTLQYY**

8 Enterobacter_sp.[WP_104948910.1] 100.0% 96.9% **REKALKAMEAERQKMQLGESYKLLEQMTNYADSPSCKEKKVCSLADAKDTFSADYEKEPGVSGPLKVGNSLVDAFTLQYY**

9 Enterobacter_vonholyi[WP_133294140.1] 100.0% 96.1% **REKALKAMETERQKMQLGESYKLLEQMTNYADSPSCKEKKVCSLADAKDTFSADYEKEPGVSGPLKVGNSLVDAFTLQYY**

10 Enterobacter_roggenkampii[WP_069598106.1] 100.0% 96.1% **REKALKAMESERQKMQLGESYKLLEQMTNYADSPSCKEKKVCSLADAKDTFSADYEKEPGVSGPLKVGNSLVDAFTLQYY**

11 Enterobacter_quasimori[WP_216357052.1] 100.0% 95.9% **REKALKAMETERQKMQLGESYKLLEQMTNYADSPSCKEKKVCSLADAKDTFSADYEKEPGVSGPLKVGNSLVDAFTLQYY**

12 Enterobacter_sp.[WP_126328197.1] 100.0% 96.1% **SEKALKAMETERQKMQLGESYKLLEQMTNYADSPSCKEKKVCSLADAKDTFSADYEKEPGVSGPLKVGNSLVDAFTLQYY**

13 Enterobacter_sp.[WP_200557788.1] 100.0% 95.4% **REKALKAMETERQKMQLSESYKLLEQMTNYADSPSCKEKKVCSLADAKDTFSADYEKEPGVSGPLKVGNSLVDAFTLQYY**

14 Enterobacter_sp.[WP_049008444.1] 100.0% 94.9% **REKALKAMEAERQKMQLGESYKLLEQMTSYADSPSCKEKKICSLADAKDTFSADYEKEPGVSGPLKVGNSLVDAFTLQYY**

15 Enterobacter_bugandensis[WP_088204980.1] 100.0% 95.2% **REKALKAMESERQKMQLGESYKLLEQMTNYADSPSCKEKKVCSLADAKDTFSADYEKEPGVSGPLKVGNSLVDAFTLQYY**

16 Enterobacter_mori[WP_237847005.1] 100.0% 95.4% **REKALKAMETERQKMQLGESYKLLEQMTNYADSPSCKEKKVCSLADAKDTFSADYEKEPGVSGPLKVGNSLVDAFTLQYY**

17 Enterobacter_pseudoroggenkampii[WP_290094731.1] 100.0% 95.4% **REKALKAMEAERQKMQLADSYKLLEQMTNYADSPSCKEKKVCSLADAKDTFSADYEKEPGVSGPLKVGNSLVDAFTLQYY**

18 Enterobacter_chuandaensis[WP_119914887.1] 100.0% 95.2% **REKALKAMETERQKMQLAESYKLLEQMTNYADSPSCKEKKVCSLADAKDTFSADYEKEPGVSGPLKVGNSLVDAFTLQYY**

19 Enterobacter_sichuanensis[WP_059385533.1] 100.0% 94.7% **RDKALKAMEAERQKMQLAESYKLLEQMTNYAESPSCKEKKVCSLADAKDTFSADYEKEPGVSGPLKVGNSLVDAFTLQYY**

20 Enterobacter_nematophilus[WP_266178332.1] 100.0% 94.4% **REKALKAMETERQKMQLGESYKLLEQMTNYVDSPSCKEKKVCSLADAKDTFSADYEKEPGVSGPLKVGNSLVDAFTLQYY**

21 Enterobacter_ludwigii[WP_040017112.1] 100.0% 93.9% **REKALKAMETERQKMQLGESYKLLEQMTNYADSPSCKEKNVCSLAEAKDTFSADYEKEPGVSGPLKVGNSLVDAFTLQYY**

22 Enterobacter_wuhouensis[WP_326471074.1] 100.0% 93.5% **REKALKAMESERQKMQLDESYRLLEQLTNYADSPSCKEKKVCSLTEAKDTFSADYEKEPGVSGPLKVGNSLVDAFTLQYY**

23 Enterobacter_huaxiensis[WP_119934760.1] 100.0% 93.2% **RDKALKAMEAERQKMQLDESYRLLEQLTNYAESPSCKEKKVCSLTEAKDTFSADYEKEPGVSGPLKVGNSLVDAFTLQYY**

24 Silvania_confinis[WP_271269508.1] 100.0% 85.5% **KEKALQAMDKERQGMHLGESYKLLEQMTDYAQSPSCKEKQVCSLSDAKDTYSADYQKEPGVSGPLKVGNSLVDAFTLQYY**

25 WP_262664223.1 100.0% 85.5% **REKALQAMNTERQGMKLDESYRLLEQMTDYKDSPSCKEKQVCSLTEAKDTYSAEYQKEPGVSGPLKVGNSLVDAFTLQYY**

26 Citrobacter_sp.[WP_045441506.1] 100.0% 84.3% **SQQAVQAMEKERSKMQLNDSYKLLEQMTNYKDSPSCKEKQQCSLIDAKDTFSAKYQQEPGVSGPLKVGNSLVDAFTLQYY**

27 Silvania_hatchlandensis[WP_271281829.1] 100.0% 84.5% **KQKALQAMDKERQGMHLGDSYKLLEKMTDYAQSPSCKEKQVCSLSEAKDTYSADYQKEPGVSGPLKVGNSLVDAFTLQYY**

28 Citrobacter_rodentium[WP_012905376.1] 100.0% 83.3% **SEQAVRAMEKERAGMQLDDSYKLLEQMTRFKDSPSCKEKQLCSLTEAKDTFSAKYQQEPGVSGPLKVGNSLVDAFTLQYY**

29 Citrobacter_farmeri[WP_042319761.1] 100.0% 83.3% **REKAVQAMEKERSQMQLDDSYKLLEQMINYKDSPSCKEKQQCSLTEAKDTFSANYEQEPGVSGPLKVGNSLVDAFTLQYY**

30 Pseudenterobacter_timonensis[WP_061708644.1] 100.0% 83.1% **HDKAISAMQAERQGMQLEESYRLLAQMTRYSDPPACKEKQVCSLTEAKDSFSADYGKEPGVSGPLKVGNSLVDAFTLQYY**

31 Citrobacter_sp.[WP_038639533.1] 100.0% 83.8% **SQQAVQAMEKERSKMQLSDSYQLLEQMTDYKDSPSCKEKQQCSLTDAKDTFSAKYQQEPGVSGPLKVGNSLVDAFTLQYY**

32 Citrobacter_freundii[WP_201506408.1] 100.0% 83.1% **SQQAVQAMEKERSKMQLNDSYQLLEQMTDYKNSPSCKEKQQCSLTDAKDTFSAKYQQEPGVSGPLKVGNSLVDAFTLQYY**

33 Pseudescherichia_vulneris[WP_154057810.1] 95.2% 86.8% **RDKAVQAMQTERQGMQLEESYKLLAQITDYADSPSCKEKQVCSLTDPKDTFSAGYEKEPGVSGPLKVGNSLVDAFTLQYY**

34 Citrobacter_telavivensis[WP_373671151.1] 100.0% 83.1% **REKAVQAMEKERSQMQLDDSYKLLEQMINYKDSPSCKEKQQCSLTEAKDTFSANDEQEPGVSGPLKVGNSLVDAFTLQYY**

35 Scandinavium_goeteborgense[WP_125354742.1] 100.0% 81.8% **SQQAVKAMSDERQKMHLGESYKLLETLVDYQNSPACKEKKVCSLSEAKDTFSAPYEKEPGVDGPLKVGNSLVDAFTLQYY**

consensus/100% **pppAlpAMptERttMpLt-SYpLLtphhpa.psPuCKEKp.CSL.-sKDoaSA..tpEPGVsGPLKVGNSLhDAFTLQYY**

consensus/90% **pppAlpAMptERptMpLs-SY+LLEQhTsYtpSPSCKEKp.CSLs-AKDTFSApYppEPGVSGPLKVGNSLVDAFTLQYY**

consensus/80% **+-KAlpAMEpERQpMQLs-SYKLLEQMTsYtDSPSCKEKpVCSLs-AKDTFSAcYpKEPGVSGPLKVGNSLVDAFTLQYY**

consensus/70% **REKAL+AMEsERQKMQLuESYKLLEQMTNYADSPSCKEKpVCSLsDAKDTFSADYEKEPGVSGPLKVGNSLVDAFTLQYY**

cov pid **241**  **: . . . . 3 . .** **320**

1 Enterobacter_cloacae_Mz49[WP_014831381.1] 100.0% 100.0% **EGFPADQVAWGEIKTDQQWRVLSKLKNGYQDSLFTSTEVAQNVAKPLVKYIDNALVTDQAKAPKITLLVGHDSNIASLLT**

2 Enterobacter_cloacae SDM [WP_014831381.1] 100.0% 100.0% **EGFPADQVAWGEIKTDQQWRVLSKLKNGYQDSLFTSTEVAQNVAKPLVKYIDNALVTDQAKAPKITLLVGHDSNIASLLT**

3 Enterobacter_cloacae[WP_248086903.1] 100.0% 99.8% **EGFPADQVAWGEIKTDQQWRVLSKLKNGYQDSLFTSTEVAQNVAKPLVKYIDNALVTDQAKAPKITLLVGHDSNIASLLT**

4 Enterobacter_cloacae[WP_335277789.1] 100.0% 99.8% **EGFPADQVAWGEIKTDQQWRVLSKLKNGYQDSLFTSTEVAQNVAKPLVKYIDNALVTDQAKAPKITLLVGHDSNIASVLT**

5 Enterobacter_cloacae[WP_368529435.1] 100.0% 99.8% **EGFPADQVAWGEIKTDQQWRVLSKLKNGYQDSLFTSTEVAQNVAKPLVKYIDNALVTDQAKAPKITLLVGHDSNIASLLT**

6 Enterobacter_cloacae[WP_262819480.1] 100.0% 99.8% **EGFPADQVAWGEIKTDQQWRVLSKLKNGYQDSLFTSTEVAQNVAKPLVKYIDNALVTDQAKAPKITLLVGHDSNIASLLT**

7 Enterobacter_cloacae[WP_230135050.1] 100.0% 99.3% **EGFPADQVAWGEVKTDQQWRVLSKLKNGYQDSLFTSTEVAQNVAKPLVKYIDNALVTDQAKAPKITLLVGHDSNIASLLT**

8 Enterobacter_sp.[WP_104948910.1] 100.0% 96.9% **EGFPADQVAWGEIKTDQQWRVLSKLKNGYQDSLFTSTEVAQNVAKPLVKYIDKTLVTDQAKAPKITLLVGHDSNIASLLT**

9 Enterobacter_vonholyi[WP_133294140.1] 100.0% 96.1% **EGFPADQVAWGEIKTDQQWRVLSKLKNGYQDSLFTSTEVAQNVAKPLVKYIDKTLVTDQAKAPKITLLVGHDSNIASLLT**

10 Enterobacter_roggenkampii[WP_069598106.1] 100.0% 96.1% **EGFPADQVAWGEIKADQQWRVLSKLKNGYQDSLFTSTEVAQNVAKPLVKYIDKTLVTEQAKAPKITLLVGHDSNIASLLT**

11 Enterobacter_quasimori[WP_216357052.1] 100.0% 95.9% **EGFPADQVAWGEIKTDQQWRVLSKLKNGYQDSLFTSTEVAQNVAKPLVKYIDKTLVTDQAKAPKITLLVGHDSNIASLLT**

12 Enterobacter_sp.[WP_126328197.1] 100.0% 96.1% **EGFPADQVAWGEIKTDQQWRVLSKLKNGYQDSLFTSTEVAQNVAKPLVKYIDKTLVTEQAKAPKITLLVGHDSNIASLLT**

13 Enterobacter_sp.[WP_200557788.1] 100.0% 95.4% **EGFPADQVAWGEIKTDQQWRVLSKLKNGYQDSLFTSTEVAQNVAKPLVKYIDKALVTDQAKAPKITLLVGHDSNIASLLT**

14 Enterobacter_sp.[WP_049008444.1] 100.0% 94.9% **EGFPADKVAWGEIKTDQQWRVLSKLKNGYQDSLFTSTEVAQNVAKPLVKYIDKALVTDQAKAPKITVLVGHDSNIASLLT**

15 Enterobacter_bugandensis[WP_088204980.1] 100.0% 95.2% **EGFPADQVAWGEIKTDQQWRVLSKLKNGYQDSLFTSTEVAQNVAKPLVKYIDKTLVTEQAKAPKITLLVGHDSNIASLLT**

16 Enterobacter_mori[WP_237847005.1] 100.0% 95.4% **EGFPADQVAWGEIKTDQQWRVLSKLKNGYQDSLFTSTEVAQNVAKPLVKYIDKTLVTEQAKAPKITLLVGHDSNIASLLT**

17 Enterobacter_pseudoroggenkampii[WP_290094731.1] 100.0% 95.4% **EGFPADRVAWGEIKTDQQWRVLSKLKNGYQDSLFTSTEVAQNVAKPLVKYIDKTLVTDQAKAPKITLLVGHDSNIASLLT**

18 Enterobacter_chuandaensis[WP_119914887.1] 100.0% 95.2% **EGFPADQVAWGEIKTDQQWRVLSKLKNGYQDSLFTSTEVAQNVAKPLVKYIDKTLVTEQAKAPKITLLVGHDSNIASLLT**

19 Enterobacter_sichuanensis[WP_059385533.1] 100.0% 94.7% **EGFPADQVAWGEIKTDQQWRVLSKLKNGYQDSLFTSTEVAQNVAKPLVKYIDKTLVTEQAKAPKITLLVGHDSNIASLLT**

20 Enterobacter_nematophilus[WP_266178332.1] 100.0% 94.4% **EGFPADQVAWGEIKTDQQWRVLSKLKNGYQDSLFTSTEVAQNVAKPLVKYIDKTLVTEQAKAPKITLLVGHDSNIASLLT**

21 Enterobacter_ludwigii[WP_040017112.1] 100.0% 93.9% **EGFPADQVAWGEIKTDQQWRVLSQLKNGYQDSLFTSTEVAQNVAKPLVKYIDQALVTGQAKAPKITLLVGHDSNIASLLT**

22 Enterobacter_wuhouensis[WP_326471074.1] 100.0% 93.5% **EGFPTDQVAWGEIKTDQQWRVLSKLKNGYQDSLFTSTDVAQNVAKPLVKYIDKALVTEQAKAPKITLLAGHDSNIASLLT**

23 Enterobacter_huaxiensis[WP_119934760.1] 100.0% 93.2% **EGFPADQVAWGEIKTDQQWRVLSKLKNGYQDSLFTSTDVAQNVAKPLVKYIDKALVTEQAKAPKITLLAGHDSNIASLLT**

24 Silvania_confinis[WP_271269508.1] 100.0% 85.5% **EGFPQDQVAWGSIKTDQQWRVLSQLKNGYQDSLFTSAEVARNVAKPLVKYIDTALVTDQAKAPKITLLVGHDSNIASLLK**

25.Leclercia tamurae strain H6S3 [WP_262664223.1] 100.0% 85.5% **EGFPLDQVAWGEIKTDQQWRVLSQLKNGYQDSLFTSAEVARNVAKPLVKYIDNALVTDQAKAPKVTLLVGHDSNIASLLK**

26 Citrobacter_sp.[WP_045441506.1] 100.0% 84.3% **EGFPMDDVAWGEIKSDQQWKVLSKLKNGYQDSLFTSPEVAQNVAKPLVKYIDNALVTERTKAPKITVLVGHDSNIASLLT**

27 Silvania_hatchlandensis[WP_271281829.1] 100.0% 84.5% **EGFPLDQVAWGEIKTDQQWRVLSQLKNGYQDSLFTSADVARNVAKPLVKYIDTALVTDQAKAPKITLLVGHDSNIASLLK**

28 Citrobacter_rodentium[WP_012905376.1] 100.0% 83.3% **EGFPMDQVAWGEIKTDQQWKVLSKLKNGYQDSLFTSVEVARNVAAPLVKYIDKALVTDRLNAPKITVLVGHDSNIASLLT**

29 Citrobacter_farmeri[WP_042319761.1] 100.0% 83.3% **EGFPMDQVAWGEIKSDQQWKVLSKLKNGYQDSLFTSAEVARNVAKPLVKYIDKTLVTEQAKAPKITVLVGHDSNIASLLS**

30 Pseudenterobacter_timonensis[WP_061708644.1] 100.0% 83.1% **EGFPMDQVAWGDINSDQQWRVLSKLKNGYQDALFTSREVAQNVAKPLVKYIDKALVTDRDKAPAITLLVGHDSNIASLLT**

31 Citrobacter_sp.[WP_038639533.1] 100.0% 83.8% **EGFPMDQVAWGEIKSDQQWKVLSKLKNGYQDSLFTSPEVAQNVAKPLVKYIDNALVTERMKAPKITVLVGHDSNIASLLT**

32 Citrobacter_freundii[WP_201506408.1] 100.0% 83.1% **EGFPMDKVAWGEIKSDQQWKVLSKLKNGYQDSLFTSPEVAQNVAKPLVKYIDNALVTERAKAPKITVLVGHDSNIASLLT**

33 Pseudescherichia_vulneris[WP_154057810.1] 95.2% 86.8% **EGFPMDQVAWGEIKTDQQWRVLSKLKNGYQDSLFTSTAVAQNVAKPLVKYIDKALVTDRASAPKVTLLVGHDSNIASLLT**

34 Citrobacter_telavivensis[WP_373671151.1] 100.0% 83.1% **EGFPMDQVAWGEIKSDQQWKVLSKLKNGYQDSLFTSAEVARNVAKPLVKYIDKTLVTEQAKAPKITVLVGHDSNIASLLS**

35 Scandinavium_goeteborgense[WP_125354742.1] 100.0% 81.8% **EGFPADQVAWGQIKTDKQWRVLSELKNGYQDSLFTSPEVARNVAAPLVKYIDKTLVTDAGKGPKVTLLVGHDSNIASLLK**

consensus/100% **EGFP.DpVAWGplpsDpQW+VLSpLKNGYQDuLFTS.tVApNVAtPLVKYIDpsLVTtt.puPtlTlLsGHDSNIASlLp**

consensus/90% **EGFPhDpVAWGEIKoDQQW+VLSpLKNGYQDSLFTSs-VApNVAKPLVKYIDpsLVT-psKAPKITlLVGHDSNIASLLp**

consensus/80% **EGFPhDQVAWGEIKTDQQWRVLSKLKNGYQDSLFTSsEVAQNVAKPLVKYIDpsLVT-QAKAPKITLLVGHDSNIASLLT**

consensus/70% **EGFPtDQVAWGEIKTDQQWRVLSKLKNGYQDSLFTSsEVAQNVAKPLVKYIDpsLVT-QAKAPKITLLVGHDSNIASLLT**

cov pid **321**  **. . : . . . . 4** **400**

1 Enterobacter_cloacae_Mz49[JBGMDT000000000] 100.0% 100.0% **ALDFKPYQLHDQQERTPIGGKIVFQRWHDKNANRELMKIEYVYQSSEQLRNASVLSLQSPAQRVTLELKGCPVDANGFCP**

2 Enterobacter_sp.[WP_014831381.1] 100.0% 100.0% **ALDFKPYQLHDQQERTPIGGKIVFQRWHDKNANRELMKIEYVYQSSEQLRNASVLSLQSPAQRVTLELKGCPVDANGFCP**

3 Enterobacter_cloacae[WP_248086903.1] 100.0% 99.8% **ALDFKPYQLHDQQERTPIGGKIIFQRWHDKNANRELMKIEYVYQSSEQLRNASVLSLQSPAQRVTLELKGCPVDANGFCP**

4 Enterobacter_cloacae[WP_335277789.1] 100.0% 99.8% **ALDFKPYQLHDQQERTPIGGKIVFQRWHDKNANRELMKIEYVYQSSEQLRNASVLSLQSPAQRVTLELKGCPVDANGFCP**

5 Enterobacter_cloacae[WP_368529435.1] 100.0% 99.8% **ALDFKPYQLHDQQERTPIGGKIVFQRWHDKNANRELMKIEYVYQSSEQLRNASVLSLQSPAQRVTLELKGCPVDANGFCP**

6 Enterobacter_cloacae[WP_262819480.1] 100.0% 99.8% **ALDFKPYQLHDQQERTPIGGKIVFQRWHDKNANRELMKIEYVYQSSEQLRNASVLSLQSPAQRVTLELKGCPVDANGFCP**

7 Enterobacter_cloacae[WP_230135050.1] 100.0% 99.3% **ALDFKPYQLHDQQERTPIGGKIVFQRWHDKNANRELMKIEYVYQSSEQLRNASVLSLQSPAQRVTLELKGCPVDANGFCP**

8 Enterobacter_sp.[WP_104948910.1] 100.0% 96.9% **ALDFKPYQLHDQQERTPIGGKIVFQRWHDKNANQELMKIEYVYQSSEQLRNASVLSLQSPAQRVTLELKGCPVDANGFCP**

9 Enterobacter_vonholyi[WP_133294140.1] 100.0% 96.1% **ALDFKPYQLHDQQERTPIGGKIVFQRWHDKNANQELMKIEYVYQSSEQLRNASVLSLQSPAQRVTLELKGCPVDANGFCP**

10 Enterobacter_roggenkampii[WP_069598106.1] 100.0% 96.1% **ALDFKPYQLHDQQERTPIGGKIVFQRWHDKNANQELMKIEYVYQSSEQLRNASVLSLQSPAQRVTLELKGCPVDANGFCP**

11 Enterobacter_quasimori[WP_216357052.1] 100.0% 95.9% **ALDFKPYQLHDQQERTPIGGKIVFQRWHDKNANRELMKIEYVYQSSEQLRNASVLSLQSPAQRVTLELRGCPVDANGFCP**

12 Enterobacter_sp.[WP_126328197.1] 100.0% 96.1% **ALDFKPYQLHDQQERTPIGGKIVFQRWHDKNANQELMKIEYVYQSSEQLRNASVLSLQSPAQRVTLELKGCPVDANGFCP**

13 Enterobacter_sp.[WP_200557788.1] 100.0% 95.4% **ALDFKPYQLHDQHERTPIGGKIVFQRWHDKNANQELMKIEYVYQSSEQLRNASVLSLESPAQRVTLELKGCPVDANGFCP**

14 Enterobacter_sp.[WP_049008444.1] 100.0% 94.9% **ALDFKPYQLHDQQERTPIGGKIVFQRWHDKNANQELMKIEYVYQSSEQLRNASVLSLQSPAQRVTLELKGCPVDANGFCP**

15 Enterobacter_bugandensis[WP_088204980.1] 100.0% 95.2% **ALDFKPYQLHDQQERTPIGGKIVFQRWHDKNSNQELMKIEYVYQSAEQLRNASVLSLQSPAQRVTLELKGCPVDANGFCP**

16 Enterobacter_mori[WP_237847005.1] 100.0% 95.4% **ALDFKPYQLHDQQERTPIGGKIVFQRWHDKNANRELMKIEYVYQSSEQLRNASVLSLQSPAQRVTLELKDCPVDANGFCP**

17 Enterobacter_pseudoroggenkampii[WP_290094731.1] 100.0% 95.4% **ALDFKPYQLHDQQERTPIGGKIVFQRWHDKNANQELMKIEYVYQSSEQLRNASVLSLQSPAQRVTLELKGCPVDANGFCP**

18 Enterobacter_chuandaensis[WP_119914887.1] 100.0% 95.2% **ALDFKPYQLHDQQERTPIGGKIVFQRWHDNNANQDLMKIEYVYQSSEQLRNASVLSLQSPAQRVTLELKGCPVDANGFCP**

19 Enterobacter_sichuanensis[WP_059385533.1] 100.0% 94.7% **ALDFKPYQLHDQQERTPIGGKIVFQRWHDKNSNQELMKIEYVYQSSEQLRNASVLSLQSPAQRVTLELKGCPVDANGFCP**

20 Enterobacter_nematophilus[WP_266178332.1] 100.0% 94.4% **ALDFKPYQLHDQQERTPIGGKIVFQRWHDKNGNQELMKIEYVYQSAEQLRNASVLSLQSPAQRVTLELKGCPVDANGFCP**

21 Enterobacter_ludwigii[WP_040017112.1] 100.0% 93.9% **ALDFKPYQLHDQHERTPIGGKIVFQRWHDKNANQELMKIEYVYQSADQLRNASVLSLDAPAQRVTLELKGCPVDANGFCP**

22 Enterobacter_wuhouensis[WP_326471074.1] 100.0% 93.5% **ALDFKTYQLHDQQERTPIGGKIVFQRWHDKNANRELMKIEYVYQSSEQLRNASVLSLESPAQRVTLELKGCPIDANGFCP**

23 Enterobacter_huaxiensis[WP_119934760.1] 100.0% 93.2% **ALDFKPYQLHDQQERTPIGGKIVFQRWHDKNASRELMKIEYVYQSSEQLRNASVLSLASPAQRVTLELKGCPIDANGFCP**

24 Silvania_confinis[WP_271269508.1] 100.0% 85.5% **ALDFKPYQLHDQYERTPIGGKIIFQRWHDKSGNRDLMKIEYVYQSTEQLRNAQVLNLKEPAQRVTLALNGCPVDGEGFCP**

25 WP_262664223.1 100.0% 85.5% **ALDFKPYQLPGQYERTPIGGKIVFQRWHDKTGNRDLMKIEYVYQSTEQLRNAQVLSLKAPAQRVTLELNGCPVDADGFCP**

26 Citrobacter_sp.[WP_045441506.1] 100.0% 84.3% **ALDFKPYQLHDQNERTPIGGKIVFQRWHDSNANRDLMKIEYVYQSSQQLRNAEVLTLKSPAQRVTLELKGCPVDTNGFCP**

27 Silvania_hatchlandensis[WP_271281829.1] 100.0% 84.5% **ALDFTPYQLHDQYERTPIGGKIIFQRWHDKTGNRDLMKIEYVYQSTEQLRNAEVLSLKDPAQRVTLALNGCPVDGEGFCP**

28 Citrobacter_rodentium[WP_012905376.1] 100.0% 83.3% **ALDFKPYQLHDQHERTPIGGKIVFQRWHDGKGNRDLMKIEYVYQSAEQLRNAEVLTLKSPAQRVTLELKGCPIDANGFCP**

29 Citrobacter_farmeri[WP_042319761.1] 100.0% 83.3% **ALEFKPYQLHDQNERTPIGGKIVFQRWHDTKANHDLMKIEYVYQSSDQLRNADVLTLKAPAQRVTLELKGCPIDANGFCP**

30 Pseudenterobacter_timonensis[WP_061708644.1] 100.0% 83.1% **ALDFTPYQLHDQYERTPIGGKIVFQRWHDKQANRDLMKIEYVYQSAQQLRNAEVLTLKSPAQRVTLSLKGCPVDANGFCP**

31 Citrobacter_sp.[WP_038639533.1] 100.0% 83.8% **ALDFKPYQLHDQNERTPIGGKIVFQRWHDSKANRDLMKIEYVYQSSQQLRNADVLTLKSPAQRVTLELKGCPVDANGFCP**

32 Citrobacter_freundii[WP_201506408.1] 100.0% 83.1% **ALDFKPYQLHDQNERTPIGGKIVFQRWHDSNANRDLMKIEYVYQSSQQLRNADVLTLKSPAQRVTLELKGCPIDADGFCP**

33 Pseudescherichia_vulneris[WP_154057810.1] 95.2% 86.8% **ALDFKPYQLHDQYERTPIGGKIVFQRWHDQAANRDLMKIEYVYQSTEQLRNADVLTLQAPAQRVTLALKGCPVDANGFCP**

34 Citrobacter_telavivensis[WP_373671151.1] 100.0% 83.1% **ALEFKPYQLHDQNERTPIGGKIVFQRWHDTKANRDLMKIEYVYQSSDQLRNADVLTLKAPAQRVTLELKGCPIDANGFCP**

35 Scandinavium_goeteborgense[WP_125354742.1] 100.0% 81.8% **ALDFKPYTLHDQYERTPIGGKIVFERWRDAAGNRELMKIEYLYQSTEQLRNADVLTLQAPPQRVTLELKGCPVDADGYCP**

consensus/100% **AL-FpsYpL.sQ.ERTPIGGKIlFpRW+Dttusp-LMKIEYlYQSspQLRNApVLsLttPsQRVTLtLpsCPlDspGaCP**

consensus/90% **ALDFKPYQLHDQ.ERTPIGGKIVFQRWHDppuNp-LMKIEYVYQSspQLRNApVLoLpuPAQRVTLpL+GCPlDAsGFCP**

consensus/80% **ALDFKPYQLHDQpERTPIGGKIVFQRWHDpsuNp-LMKIEYVYQSoEQLRNAsVLoLpuPAQRVTLELKGCPVDANGFCP**

consensus/70% **ALDFKPYQLHDQpERTPIGGKIVFQRWHDKNANp-LMKIEYVYQSSEQLRNAsVLSLpSPAQRVTLELKGCPVDANGFCP**

cov pid **401**  **. ]** **413**

1 Enterobacter_cloacae_Mz49[JBGMDT000000000] 100.0% 100.0% **IDKFNTVMNDAAK**

2 Enterobacter_sp.[WP_014831381.1] 100.0% 100.0% **IDKFNTVMNDAAK**

3 Enterobacter_cloacae[WP_248086903.1] 100.0% 99.8% **IDKFNTVMNDAAK**

4 Enterobacter_cloacae[WP_335277789.1] 100.0% 99.8% **IDKFNTVMNDAAK**

5 Enterobacter_cloacae[WP_368529435.1] 100.0% 99.8% **IDKFNTVMNDAAK**

6 Enterobacter_cloacae[WP_262819480.1] 100.0% 99.8% **IDKFNTVMNDAAK**

7 Enterobacter_cloacae[WP_230135050.1] 100.0% 99.3% **IDKFNTVMNDAAK**

8 Enterobacter_sp.[WP_104948910.1] 100.0% 96.9% **VDKFNAVMNNAAK**

9 Enterobacter_vonholyi[WP_133294140.1] 100.0% 96.1% **VDKFNAVMNNAAK**

10 Enterobacter_roggenkampii[WP_069598106.1] 100.0% 96.1% **VDKFNAVMNNAAK**

11 Enterobacter_quasimori[WP_216357052.1] 100.0% 95.9% **VDKFNAVMNNAAK**

12 Enterobacter_sp.[WP_126328197.1] 100.0% 96.1% **VDKFNAVMNNAAK**

13 Enterobacter_sp.[WP_200557788.1] 100.0% 95.4% **VDKFNAVMNSAAR**

14 Enterobacter_sp.[WP_049008444.1] 100.0% 94.9% **VDKFNAVINNAAK**

15 Enterobacter_bugandensis[WP_088204980.1] 100.0% 95.2% **VDKFNAVMNNAAK**

16 Enterobacter_mori[WP_237847005.1] 100.0% 95.4% **VDKFNAVMNNAAK**

17 Enterobacter_pseudoroggenkampii[WP_290094731.1] 100.0% 95.4% **VDKFNAVMNNAAK**

18 Enterobacter_chuandaensis[WP_119914887.1] 100.0% 95.2% **VDKFNAVMNNAAK**

19 Enterobacter_sichuanensis[WP_059385533.1] 100.0% 94.7% **VDKFNAVMNNAAK**

20 Enterobacter_nematophilus[WP_266178332.1] 100.0% 94.4% **VDKFNAVMNNAAK**

21 Enterobacter_ludwigii[WP_040017112.1] 100.0% 93.9% **IDKFNAVMNNAAK**

22 Enterobacter_wuhouensis[WP_326471074.1] 100.0% 93.5% **IDKFNAVMNDAAK**

23 Enterobacter_huaxiensis[WP_119934760.1] 100.0% 93.2% **IDKFNAVMNDAAK**

24 Silvania_confinis[WP_271269508.1] 100.0% 85.5% **IETFNTVLNTAAK**

25 WP_262664223.1 100.0% 85.5% **VETFNTLLNTAAK**

26 Citrobacter_sp.[WP_045441506.1] 100.0% 84.3% **IDKFDSVLNEAAK**

27 Silvania_hatchlandensis[WP_271281829.1] 100.0% 84.5% **IETFNKVLNAAAK**

28 Citrobacter_rodentium[WP_012905376.1] 100.0% 83.3% **IDKFDALLNEAAK**

29 Citrobacter_farmeri[WP_042319761.1] 100.0% 83.3% **IDKFDTLLNAAAK**

30 Pseudenterobacter_timonensis[WP_061708644.1] 100.0% 83.1% **MDKFTMVMNEAAK**

31 Citrobacter_sp.[WP_038639533.1] 100.0% 83.8% **IDKFDSVLNEAAK**

32 Citrobacter_freundii[WP_201506408.1] 100.0% 83.1% **IDKFDSVLNEAAK**

33 Pseudescherichia_vulneris[WP_154057810.1] 95.2% 86.8% **VDKFDAVLNDAAK**

34 Citrobacter_telavivensis[WP_373671151.1] 100.0% 83.1% **IDKFDALLNEAAK**

35 Scandinavium_goeteborgense[WP_125354742.1] 100.0% 81.8% **VDKFNEVLNNAAK**

consensus/100% **h-pFs.lhNtAA+**

consensus/90% **lDKFsslhNpAAK**

consensus/80% **lDKFssVhNsAAK**

consensus/70% **lDKFNsVhNsAAK**

**Fig. S7. Multiple sequence alignment of the predicted amino acid sequences of *agp* in Mz49 compared to that of the closely related proteins retrieved from the NCBI database.** The alignment was visualized using MView version 1.63; cov, coverage; pid, percent identity. The conserved domains of active sites and catalytic core are illustrated in the black box, where RH (Residue 39,40), R (Residue 43), R (Residue 116), and HD (Residue 311,312). The red box represents the histidine acid phosphatases phosphohistidine signature domain LeqVliMsRHNlRaP (Residue 31-45).

1 . . . . . . . . 10 . . . . . . . . . 20 . . . . . . . . . 30 . . . . . . . . . 40 . . . . . . . . . 50 60

Sequence M R K A L L A V A V A G L F S A A S G V Q A Q E T P E G Y Q L E Q V L I M S R H N L R A P L A N N G S V L E Q S T P K Q


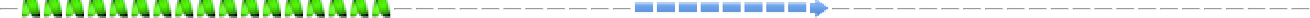
Secondary structure

SS


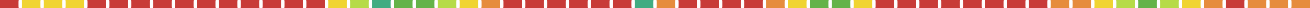


confidence

Disorder **? ?** - - - - - - - - - - - - - - - - - - - - - **? ? ? ? ? ?** - - - - - - - - - - - - - - - - - - - - - - - - - - - - - - -


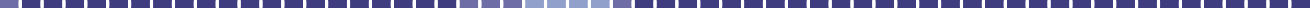
Disorder confidence

. . . . . . . . . 70 . . . . . . . . . 80 . . . . . . . . . 90 . . . . . . . . . 100 . . . . . . . . . 110 120

Sequence W P E W E V P G G Q L T T K G G V L E V Y M G H Y M R E W L A Q Q G M V K T G E C P P A E S V Y A Y A N S L Q R T V A T


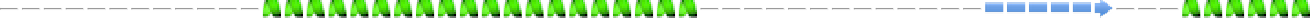
Secondary structure

SS


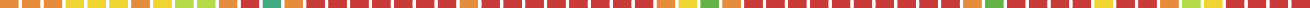


confidence

Disorder - - - - - - - - - - - - - - - - - - - - - - - - - - - - - - - - - - - - - - - - - - - - - - - - - - - - - - - - - - - -


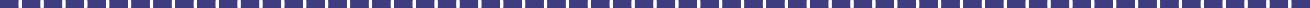
Disorder confidence

. . . . . . . . . 130 . . . . . . . . . 140 . . . . . . . . . 150 . . . . . . . . . 160 . . . . . . . . . 170 180

Sequence A Q F F I T G A F P G C D V P V H H Q D K M G T M D P T F N P V I T D N S P E F R E K A L K A M E S E R Q K M Q L G E S


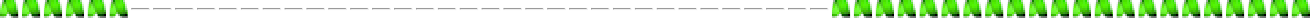
Secondary structure

SS


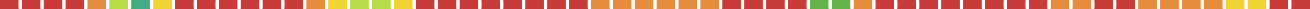


confidence

Disorder - - - - - - - - - - - - - - - - - - - - - - - - - - - - - - - - - - - - - - - - - - - - - - - - - - - - - - - - - - - -


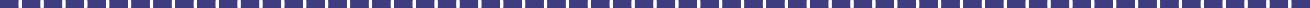
Disorder confidence

. . . . . . . . . 190 . . . . . . . . . 200 . . . . . . . . . 210 . . . . . . . . . 220 . . . . . . . . . 230 240

Sequence Y K L L E Q M T N Y A D S P S C K E K K V C S L A D A K D T F S A D Y E K E P G V S G P L K V G N S L V D A F T L Q Y Y


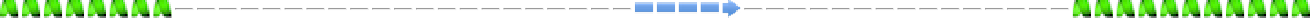
Secondary structure

SS


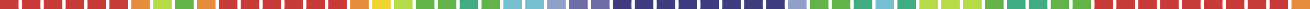


confidence

Disorder - - - - - - - - - - - - - - - - - - - - - - - - - - - - - - - - - - - - - - - - - - - - - - - - - - - - - - - - - - - -


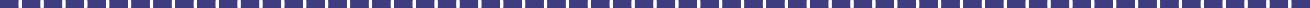
Disorder confidence

. . . . . . . . . 250 . . . . . . . . . 260 . . . . . . . . . 270 . . . . . . . . . 280 . . . . . . . . . 290 300

Sequence E G F P A D Q V A W G E I K T D Q Q W R V L S K L K N G Y Q D S L F T S T E V A Q N V A K P L V K Y I D N A L V T D Q A


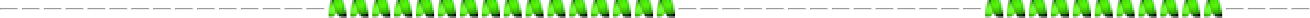
Secondary structure

SS


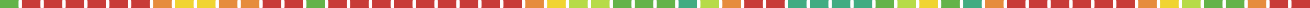


confidence

Disorder - - - - - - - - - - - - - - - - - - - - - - - - - - - - - - - - - - - - - - - - - - - - - - - - - - - - - - - - - - - -


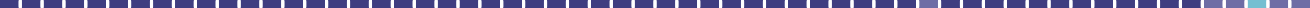
Disorder confidence

. . . . . . . . . 310 . . . . . . . . . 320 . . . . . . . . . 330 . . . . . . . . . 340 . . . . . . . . . 350 360

Sequence K A P K I T L L V G H D S N I A S L L T A L D F K P Y Q L H D Q Q E R T P I G G K I V F Q R W H D K N A N R E L M K I E


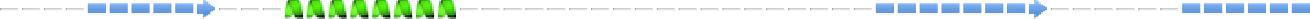
Secondary structure

SS


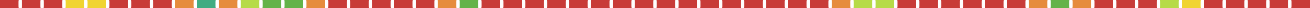


confidence

Disorder - - - - - - - - - - - - - - - - - - - - - - - - - - - - - - - - - - - - - - - - - - - - - - - - - - - - - - - - - - - -


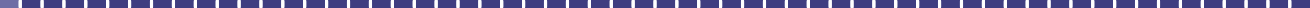
Disorder confidence

. . . . . . . . . 370 . . . . . . . . . 380 . . . . . . . . . 390 . . . . . . . . . 400 . . . . . . . . . 410 . . .

Sequence Y V Y Q S S E Q L R N A S V L S L Q S P A Q R V T L E L K G C P V D A N G F C P I D K F N T V M N D A A K


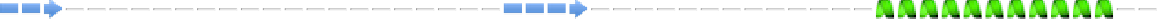
Secondary structure

SS


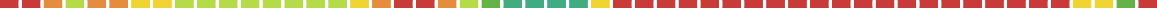


confidence

Disorder **?**


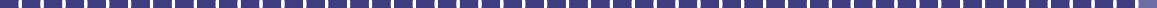
Disorder confidence

Confidence Key

High(9)
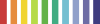
 Low (0)

**?** Disordered ( 2%)
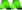
 Alpha helix ( 33%)

**Fig. S8. Predicted Secondary structure and disorder prediction of the phytase protein from Mz49 generated using Phyre2.** The analysis indicates 33% alpha-helices, 11% beta-strands, and 2% disordered regions, suggesting a predominantly ordered structure with defined folding regions. Confidence scores across the sequence reflect high reliability for the predicted structural elements.


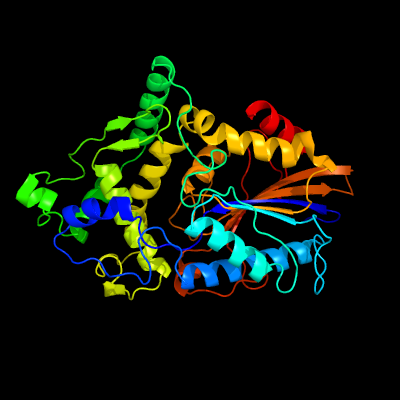


**Fig. S9. Predicted tertiary structure of the phytase protein from Mz49 generated using Phyre2 prediction tool.** The figure showss various elements of protein secondary structure, including alpha helices (colored in shades of green, yellow, and red) and beta sheets (represented in blue and cyan), arranged in a compact fold essential for catalytic activity. This structural organization supports substrate binding and hydrolysis of phytate.
